# First description of epimorphic development in Antarctic Pallenopsidae (Arthropoda, Pycnogonida) with insights into the evolution of the four-articled sea spider cheliphore

**DOI:** 10.1186/s40851-018-0118-7

**Published:** 2019-01-14

**Authors:** Georg Brenneis, Claudia P. Arango

**Affiliations:** 1 Zoologisches Institut und Museum, Cytologie und Evolutionsbiologie, Universität Greifswald, Soldmannstraße 23, 17489 Greifswald, Germany; 2 Institut für Biologie, Vergleichende Zoologie, Humboldt-Universität zu Berlin, Philippstraße 13, Haus 2, 10115 Berlin, Germany; 3 Queensland Museum, Biodiversity Program, PO Box 3300, South Brisbane, QLD 4101 Australia

**Keywords:** *Pallenopsis*, East Antarctica, Postembryonic development, Direct development, Ontogeny, Morphogenesis, Evolution, Phylogeography, Fossils, Scanning electron microscopy, Fluorescent histochemistry

## Abstract

**Background:**

Sea spiders (Pycnogonida) are an abundant faunal element of the Southern Ocean (SO). Several recent phylogeographical studies focused on the remarkably diverse SO pycnogonid fauna, resulting in the identification of new species in previously ill-defined species complexes, insights into their genetic population substructures, and hypotheses on glacial refugia and recolonization events after the last ice age. However, knowledge on the life history of many SO pycnogonids is fragmentary, and early ontogenetic stages often remain poorly documented. This impedes assessing the impact of different developmental pathways on pycnogonid dispersal and distributions and also hinders pycnogonid-wide comparison of developmental features from a phylogenetic-evolutionary angle.

**Results:**

Using scanning electron microscopy (SEM) and fluorescent nuclear staining, we studied embryonic stages and postembryonic instars of three SO representatives of the taxon Pallenopsidae (*Pallenopsis villosa*, *P. hodgsoni*, *P. vanhoeffeni*), the development of which being largely unknown. The eggs are large and yolk-rich, and the hatching stage is an advanced lecithotrophic instar that stays attached to the father for additional molts. The first free-living instar is deduced to possess at least three functional walking leg pairs. Despite gross morphological similarities between the congeners, each instar can be reliably assigned to a species based on body size, shape of ocular tubercle and proboscis, structure of the attachment gland processes, and seta patterns on cheliphore and walking legs.

**Conclusions:**

We encourage combination of SEM with fluorescent markers in developmental studies on ethanol-preserved and/or long term-stored pycnogonid material, as this reveals internal differentiation processes in addition to external morphology. Using this approach, we describe the first known cases of pallenopsid development with epimorphic tendencies, which stand in contrast to the small hatching larvae in other Pallenopsidae. Evaluation against current phylogenetic hypotheses indicates multiple gains of epimorphic development within Pycnogonida. Further, we suggest that the type of development may impact pycnogonid distribution ranges, since free-living larvae potentially have a better dispersal capability than lecithotrophic attaching instars. Finally, we discuss the bearing of pycnogonid cheliphore development on the evolution of the raptorial first limb pair in Chelicerata and support a multi-articled adult limb as the plesiomorphic state of the chelicerate crown group, arising ontogenetically via postembryonic segmentation of a three-articled embryonic limb.

**Electronic supplementary material:**

The online version of this article (10.1186/s40851-018-0118-7) contains supplementary material, which is available to authorized users.

## Introduction

The exclusively marine sea spiders (Pycnogonida) show extraordinary species diversity and high abundances in the Southern Ocean (SO), making them an important benthic faunal element especially on the Antarctic shelf (e.g., [1]). Representatives of all major pycnogonid taxa (“families”) are recorded in Antarctic and Subantarctic waters, comprising in total no less than 20% of all described species (264 out of 1344 species in 2009), and a substantial number of them is endemic to these regions [2, 3]. Moreover, new species continue to be described on a regular basis. This is due to new material from previously poorly sampled areas (e.g., [4, 5]) and a growing body of molecular phylogeographical analyses, which have started to reassess and disentangle notoriously variable pycnogonid “species” with wide distribution ranges (e.g., [3, 6–11]). Apart from the identification of previously unrecognized species, these studies have also given insights into patterns of glacial refugia and post-glaciation (re)colonization events of the diverse SO pycnogonid fauna (e.g., [3, 12, 13]).

Beyond integrative taxonomy and phylogeography, several recent studies have provided details on the postembryonic development of SO pycnogonids [14–19]. Sea spiders show paternal brood care; males carry egg batches and/or postembryonic instars on their modified third limb pair, the ovigers. Interestingly, however, different pycnogonid taxa exhibit different types of development (see [20] for recent review). The most common pycnogonid hatching stage is a small protonymphon larva with three limb pairs that correspond to the first three adult limbs, i.e., the cheliphore, palp and aforementioned oviger. Posterior to the latter, the larval body comprises one or two internally—but not yet externally—recognizable segment primordia (e.g., [21, 22]). Only during the free-living and parasitic postembryonic phase the remaining trunk segments are added and further developed, which is externally discretized by anamorphic molts (type 1 development sensu [20]; see, e.g., [23]). In contrast to this, some taxa follow deviating developmental pathways. These may feature, e.g., lecithotrophic nutrition of significantly larger protonymphon larva and attachment of postlarval instars to the paternal oviger (type 2 development; e.g., [14, 15, 24]), or even a (partial) shift of trunk differentiation into the embryonic phase, resulting in the hatching of significantly advanced postlarval instars (type 5 development; e.g., [25–28]). So far, these developmental differences have been given only cursory attention in phylogeographical contexts, where pycnogonids are very generally treated as “brooders” (i.e., eggs are carried by males until hatching) and “crawlers” (i.e., no pelagic stage in the life cycle) with limited dispersal potential [1, 3]. However, comprehensive comparison and potential correlation of developmental types with distribution ranges in the SO and beyond are lacking. To a large extent, this is due to sparse or completely missing data on the development of many species, impeding meaningful appraisal of the effect of the developmental type on pycnogonid dispersal capability. Further, this paucity of developmental data precludes its inclusion in phylogenetic analyses and evolutionary considerations, not only within Pycnogonida but also in an arthropod-wide framework.

To help address this lack of data, we studied embryonic and postembryonic stages of Antarctic representatives of Pallenopsidae, a pycnogonid taxon for which only the protonymphon larvae of three species are known so far [29, 30]. Using scanning electron microscopy (SEM) and fluorescent nuclear staining, we describe herein the first cases of pallenopsid development with epimorphic tendencies and evaluate this novel developmental mode for Pallenopsidae relative to current hypotheses on pycnogonid phylogeny. Further, we discuss the impact of different developmental types on the dispersal potential of different pycnogonid species. Finally, we highlight the bearing of pallenopsid cheliphore development on our understanding of the evolution of the raptorial first limb pair in Chelicerata.

## Material and methods

### Material collection and species identification

All embryonic and postembryonic developmental stages were taken from ovigerous pallenopsid males collected during the CEAMARC 2007/2008 cruise to the Dumont d’Urville Sea, East Antarctica (details in [31]). Specimens were collected from the seafloor by beam trawl, sorted onboard and immediately preserved in 90% ethanol upon collection. The sampling dates, locations and water depths are listed in Table 1.

**Table 1:** Species, collection data and developmental stage of material studied

Species	Sample no.	Sampling location [°S/°E]	Sampling date	Depth [m]	Stage
*Pallenopsis villosa* Hodgson, 1907	IU-2007-71d IU-2007-283	66.143585/143.295548 66.753423/145.208488	04. Jan. 2008 30. Dec. 2007	534 568–597	germ disc/band hatched instar 1
*Pallenopsis hodgsoni* Gordon, 1938	IU-2007-32 IU-2007-114	65.869947/143.001547 66.333008/140.652127	04. Jan. 2008 14. Jan. 2008	428–430 165–168	instar 2 prehatching embryo & hatching instar 1
*Pallenopsis vanhoeffeni* Hodgson, 1915	IU-2007-40e	66.38878/140.428852	14. Jan. 2008	791	instar 2

Identification of pallenopsid species followed the detailed key for Antarctic and Subantarctic pycnogonids by Child [32]. Importantly, this key recognizes *Pallenopsis hodgsoni* as a separate species and distinguishes it from the similar congener *P. pilosa*. This distinction is not universally accepted (e.g., [2]), but an integrative taxonomic reinvestigation of this potential species complex is pending. Based on the set of diagnostic characters listed by Child [32], we here retain the species delimitation of *P. hodgsoni* and *P. pilosa* (see also [5, 33]) and assigned part of the material studied to the former.

Egg packages and attaching posthatching instars were carefully removed from the males’ ovigers using soft forceps. If still enclosed by the elastic matrix of the egg package, developmental stages were manually freed with dissecting needles.

### Fluorescent nuclear staining, data documentation and analysis

As direct fixation of pycnogonid developmental stages in 90% ethanol often introduces morphological artifacts in body regions with weakly sclerotized cuticle (e.g., collapse and artificial folding of appendages), some specimens were rehydrated via a graded ethanol series into double-distilled water (ddH_2_O). In several specimens, this treatment led to an unfolding of previously collapsed structures. Subsequently, nuclear staining with the fluorescent marker Sytox®Green (Invitrogen Molecular Probes®, 1:1000 in ddH_2_O) was performed overnight at 4 °C. This staining not only allows documentation of the gross external morphology, but also provides more insights into the differentiation stage of internal structures, such as leg podomeres or ganglia of the central nervous system.

Z-stacks of the Sytox®Green-stained specimens were taken with a Zeiss Lumar V12 and automatically aligned with Zeiss AxioVision software (Version 4.7.10). Each aligned z-stack was subsequently merged to a single image with extended depth of field using Helicon Focus software (Heliconsoft, Version 6.7.1).

### Scanning electron microscopy (SEM)

Embryos and postembryonic instars were dehydrated via a graded ethanol series (90, 96%, 2 × 100%; each step at least 30 min), critical point-dried with a Bal-Tec CPD 030, and sputtered with gold using a Bal-Tec SCD 005. Micrographs were taken with a Zeiss LEO 1430 scanning electron microscope.

### Applied terminology and data presentation

Designation of the pallenopsid postembryonic stages as postlarval instars follows the definitions recently suggested by Brenneis and colleagues [20]. We consider the “main claw” of the walking legs as the ultimate podomere, which forms a subchelate complex with the propodus. Consequently, the adult leg article count is nine (see also [34, 35]). Walking leg podomeres are numbered from proximal to distal in the provided images (i.e., coxae 1–3 = 1–3, respectively; femur = 4; tibiae 1 & 2 = 5 & 6, respectively; tarsus = 7; propodus = 8; main claw = 9). Body length measurements were performed on SEM micrographs and epifluorescence images as a straight line between the anterior tip of the body to its posterior end (anal tubercle, if present). The cheliphores were not included in the measurement.

Global contrast and brightness values of images were adjusted using Adobe Photoshop CS5. All figures were compiled with Adobe Illustrator CS5. If not stated otherwise, anterior is (1) toward the top in ventral or dorsal views and (2) toward the left in lateral views.

## Results

### *Pallenopsis villosa* Hodgson, 1907

Two ovigerous males were found in the CEAMARC 2007/2008 material (Table 1). One male had both ovigers inserted into a single voluminous egg package, the other carried attaching postlarval instars.

#### Embryonic stages (nuclear stain *n* = 9; Fig. 1)

**Fig. 1:**
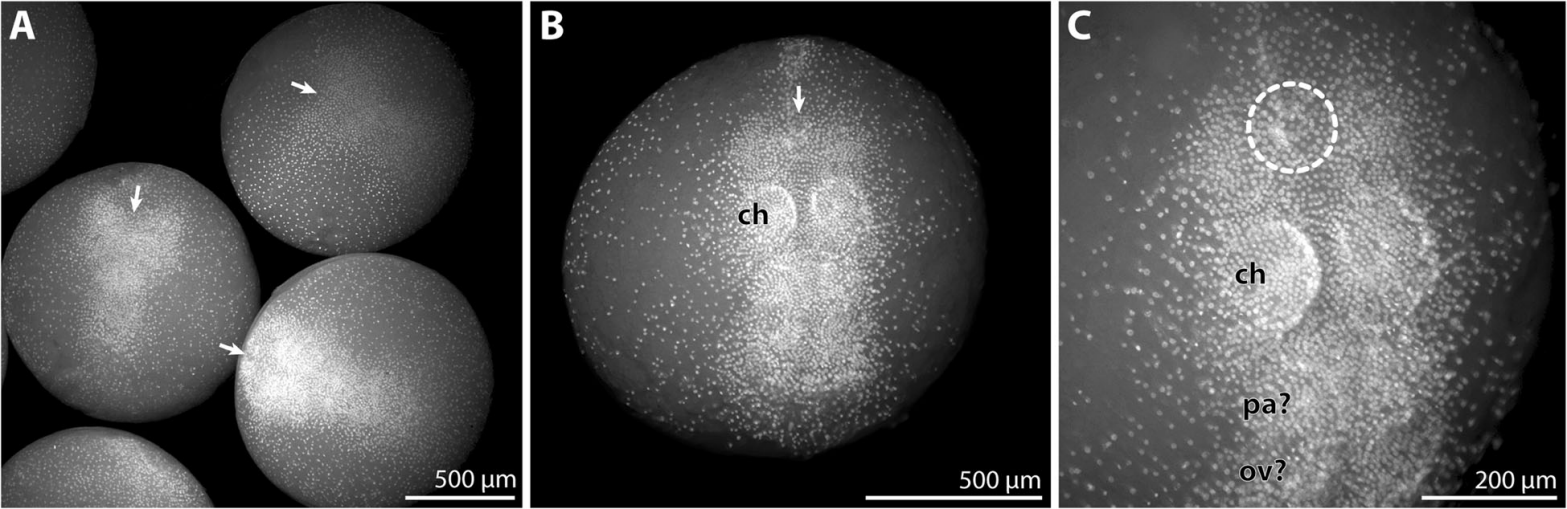
Embryonic stages of *Pallenopsis villosa*. Stereomicroscope images of Sytox-stained embryos. Arrows mark the anterior pole of the germ band. **a** Late germ disc and early germ band stages. Note the distinctly higher density of nuclei in the most advanced stage in the lower right corner. **b** Most advanced embryonic stage in the material studied. Ventral view. The primordia of the cheliphore are visible as small protrusions in the anterior half of the germ band. **c** Higher magnification of the embryo shown in **b**. Ventro-lateral view. The stippled circle highlights the area of the invaginating stomodeum. The palpal and ovigeral segment anlagen (pa? and ov?, respectively) are likely to be defined at this stage, but distinct limb primordia are still absent. Abbreviations: ch – cheliphore; ov? – ovigeral segment anlage?; pa? – palpal segment anlage?

The yolk-rich eggs of *P. villosa* are of spherical shape and considerable size, with a diameter of slightly more than 1 mm (Fig. 1a, b). Fluorescent nuclear staining revealed an early phase of morphogenesis. Development within the egg package is not completely synchronous, as some of the embryos were found to be slightly more advanced than others (Fig. 1a, b).

The earliest stages observed are in a late germ disc stage, featuring an agglomeration of embryonic cells in one hemisphere of the egg (Fig. 1a). The germ disc covers only a small fraction of the hemisphere, but with ongoing development shows an increase in cell numbers and an elongation into a germ band (Fig. 1a, b). The slightly wider side of the germ band represents the prospective anterior body pole, whereas the opposite, narrower side is the area in which the anlagen of more posterior body segments are being formed (Fig. 1a). In the most advanced stages available, invagination of the stomodeum has been initiated at the anterior pole and the cheliphoral limb buds have started to appear posterior to it (Fig. 1b, c). It remains unclear how many body segments are prefigured in the embryonic germ band posterior to the cheliphoral segment, as no further limb buds are formed at this stage and older embryos were lacking.

#### Postlarval instar 1 (SEM *n* = 7, nuclear stain *n* = 7; Figs. 2 and 3; Table 2)

**Fig. 2:**
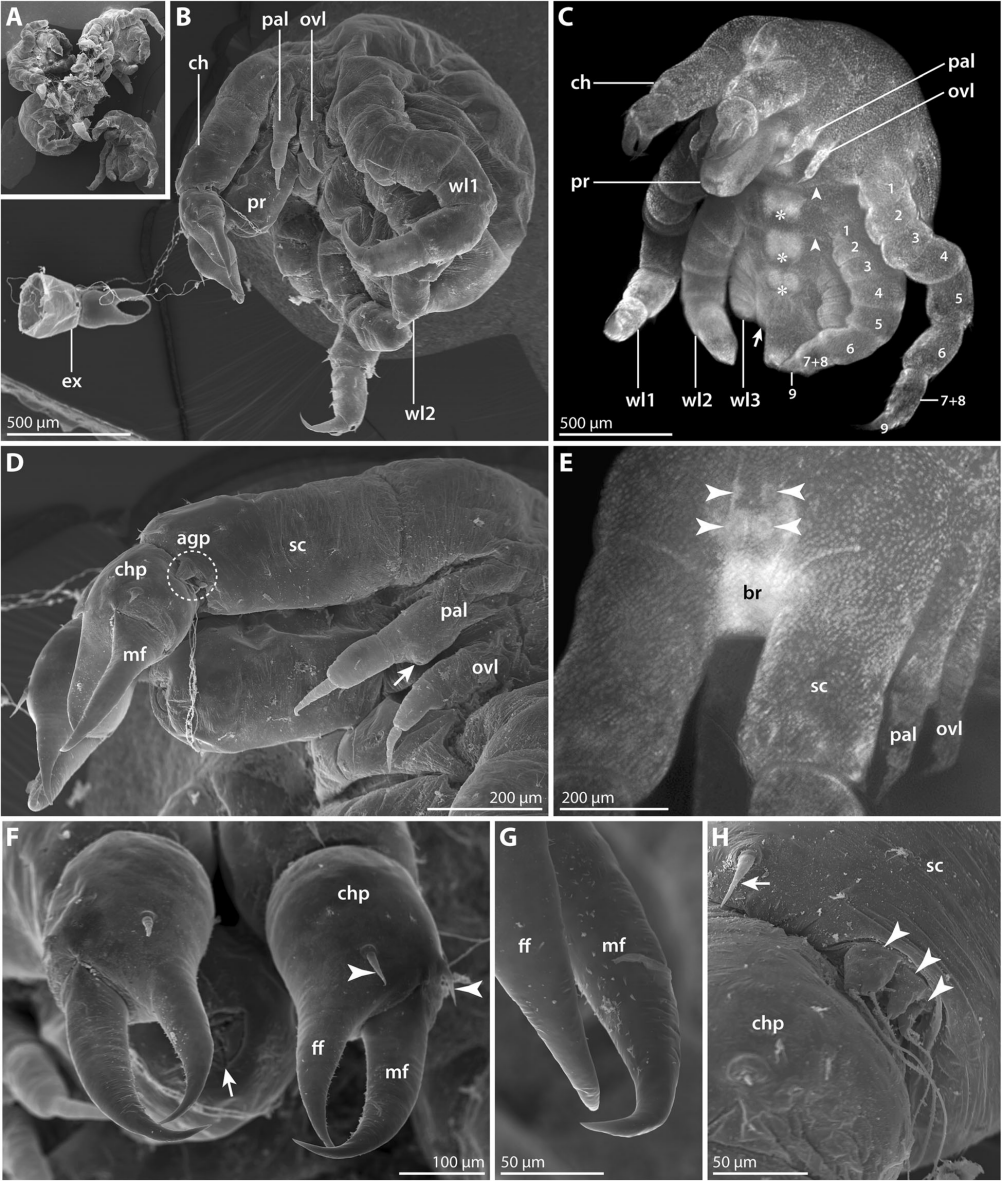
Postlarval instar 1 of *Pallenopsis villosa*. SEM micrographs (**a**, **b**, **d**, **f**-**h**) and stereomicroscopic images of Sytox-stained specimens (**c**, **e**). **a** Overview of four specimens still attached to a piece of egg matrix. **b** Lateral view. Note the cheliphoral exuvia that remains strung on the fibrous attachment gland secretion. **c** Ventrolateral view. Note the developing podomeres (1–9) in walking leg 1 but also in the externally unarticulated walking leg 2. The arrow points at the minute primordium of walking leg 4. Asterisks mark the growing ganglion anlagen of the VNC. Arrowheads indicate ventral intersegmental folds. **d** Detail of the anterior body pole, lateral view. The stippled circle highlights the short attachment gland processes on the scape. The arrow points at the posterior protrusion on the proximal article of the palpal larval limb. **e** Detail of the anterior body pole, anterolateral view. Arrowheads point at the four eye anlagen dorsal to the brain. **f** Detail of the chelae. Arrowheads indicate the setae at the dorsal base of each chela finger. The arrow indicates the Y-shaped mouth which is surrounded by three cuticular lips. **g** Detail of the chela finger tips which lack (sub)terminal pores, indicating the absence of chela glands. **h** Detail of the scape’s distal end. Arrowheads point at the multiple attachment gland processes with emanating fibrous secretion. The arrow highlights one of the two dorsal setae on the scape. Abbreviations: agp – attachment gland process; br – brain; ch – cheliphore; chp – chela palm; ex – chela exuvia; ff – fixed chela finger; mf – moveable chela finger; ovl – ovigeral larval limb; pal – palpal larval limb; pr – proboscis; sc – scape; wl – walking leg

Numerous specimens belonging to this instar were attached to the ovigers of the second *P. villosa* male (Fig. 2a). The instar is lecithotrophic and contains a copious yolk supply. Its body is of ovoid shape, measuring 1.5 mm or more along the anterior-posterior (a-p) axis (Fig. 2b, c).

**Fig. 3:**
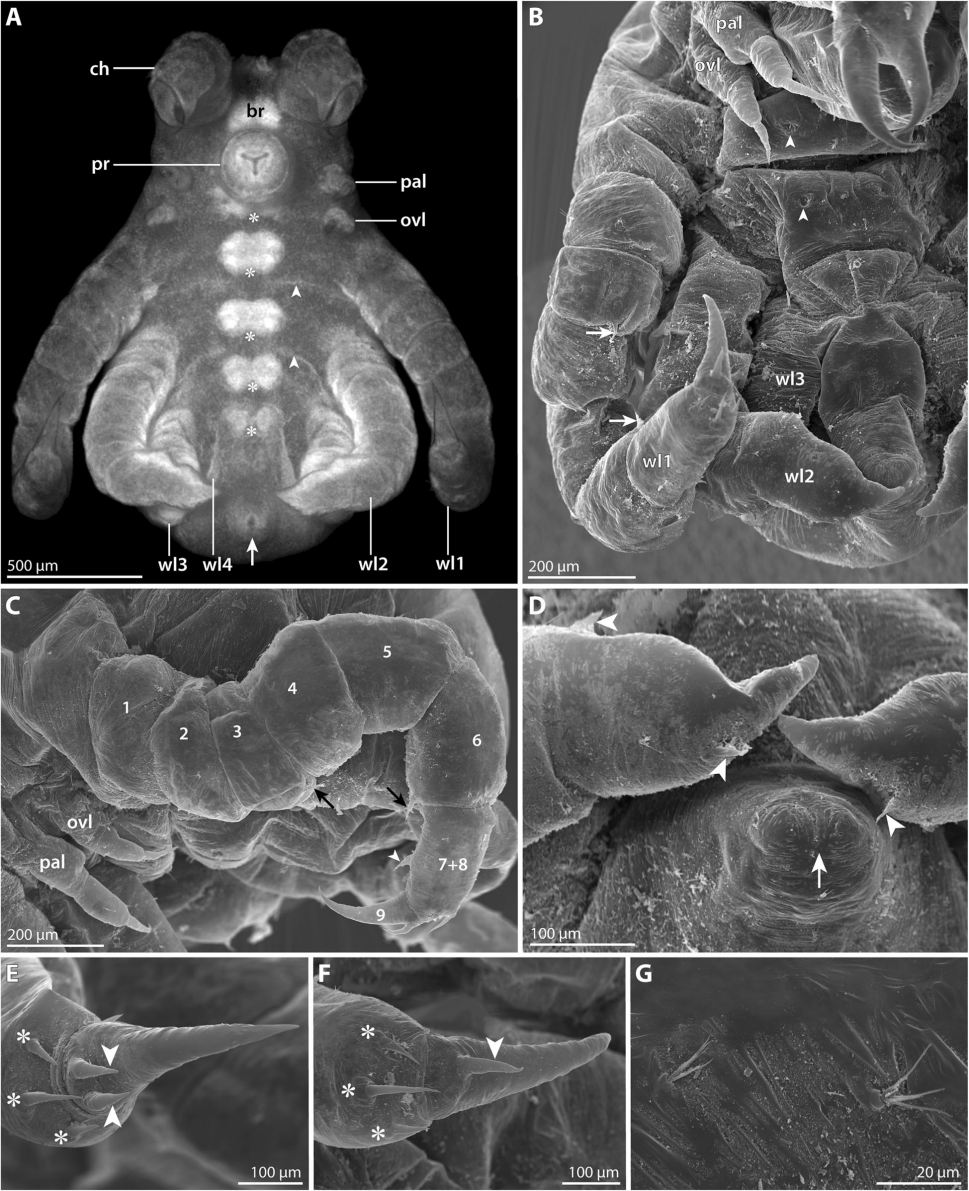
Postlarval instar 1 of *Pallenopsis villosa* (continued). SEM micrographs (**b–g**) and stereomicroscopic image of Sytox-stained specimen (**a**). **a** Ventral view. Asterisks mark the developing ventral ganglia. The arrow points at the slit-shaped proctodeum anlage. Arrowheads indicate ventral intersegmental folds between the walking leg segments. **b** Detail in ventral view. Arrowheads mark the cuticular pits of the ventral organs in walking leg segments 1 and 2. Arrows point at the ventro-distal setae on coxa 3 and tibia 2 of walking leg 1. **c** Lateral view of walking leg 1. Note complete external articulation apart from the tarsus-propodus precursor (7 + 8). Arrows point at the ventro-distal setae on coxa 3 and tibia 2. The arrowhead highlights a ventral spine/seta on the tarsus-propodus precursor. **d** Detail of the posterior body pole. The cuticle of the anal tubercle is distally closed (arrow). Arrowheads indicate the characteristic ventro-distal seta of future tibia 2 and the subterminal seta marking the distal end of the prospective propodus. **e** & **f** Details of the main claw of walking leg 1. The asterisks mark the three stereotypic setae at the distal end of the future propodus. Note the presence either of two (**e**) or only one (**f**) auxiliary claw(s) (arrowheads) at the base of the main claw. **g** Detail of the bifurcating and trifurcating “Gabelborsten” types. Abbreviations: br – brain; ch – cheliphore; ovl – ovigeral larval limb; pal – palpal larval limb; pr – proboscis; wl – walking leg

**Table 2:** Similarities and differences between postlarval instar 1 of the pallenopsids studied

Instar 1 (hatching stage)	Species		
	*P. villosa*	*P. hodgsoni*	*P. vanhoeffeni*
Nutrition	lecithotrophic	lecithotrophic	lecithotrophic
Size (a-p axis in mm)	**≥ 1.5**	**1.0–1.2**	?
Ocular tubercle	not yet elevated	not yet elevated	?
Eyes	anlagen present	anlagen present	?
Proboscis	**barrel-shaped**	**bullet-shaped**	?
Cheliphore	3-articled	3-articled	?
Chela fingers	**evenly curved toward tip**	**proximal part +/− straight, then curving toward tip**	?
Attachment gland process	**multiple** & short	**single** & short	?
Palpal larval limb	3-articled; **>ovigeral limb**	3-articled **> > ovigeral limb**	?
Ovigeral larval limb	“3-articled” → articles 1&2 not well-defined	“3-articled” → articles 1&2 not well-defined	?
Walking leg 1	externally 8-articled; **internally propodus & tarsus not yet separated**	externally 8-articled; **internally propodus and tarsus distinctly separated**	elongate – structural details unknown
Walking leg 2	elongate limb bud; **internally 8 articles defined**	elongate limb bud; **internally 9 articles defined**	elongate – structural details unknown
Walking leg 3	tiny elevation w/ compressed internal tissue	tiny elevation w/ compressed internal tissue	tiny elevation w/ compressed internal tissue
Walking leg 4	internal primordium; externally not recognizable	internal primordium; externally not recognizable	?
Anal tubercle	w/ slit-shaped proctodeum; anus not open yet	w/ slit-shaped proctodeum; anus not open yet	anus not open yet
Ventral side of trunk	w/ intersegmental folds	?	?
Dorsal side of trunk	no signs of segmentation	no signs of segmentation	no signs of segmentation
Central nervous system	all ventral ganglion anlagen present ; distinct a-p gradient	all ventral ganglion anlagen present ; distinct a-p gradient	?

Main differences between species are highlighted in bold. In case of lack of or insufficient data (see especially *P. vanhoeffeni*), questions marks are shown

An ocular tubercle is not yet evident (Fig. 2b, c), but nuclear staining reveals two pairs of developing eyes underneath the cuticle (Fig. 2e). Owing to the material’s preservation in ethanol, it is unclear whether these eye anlagen express shading pigments in live specimens.

The proboscis is directed ventrally. It is barrel-shaped, lacking constrictions along the proximo-distal (p-d) axis (Fig. 2c, d). Distally, it tapers slightly towards the terminally located mouth that is surrounded by three cuticular lips lending the mouth its characteristic Y-shape (Figs. 2c, f and 3a).

The cheliphore flanks the proboscis antero-laterally, protruding in a ventral direction. It is comprised of the undivided proximal scape plus the second and third articles forming a chela (Fig. 2b-d).

The scape is of the same length as the proboscis (Fig. 2d). Distally, it bears two dorsal setae (Fig. 2f, h) and a lateral group of short processes, each of them bearing at least one distal pore from which fibrous secretions of the cheliphoral attachment gland protrude (Fig. 2d, h). The number of processes varies among specimens, ranging from two to four. In many specimens, a cheliphore exuvia was still strung on the secreted fibers (Fig. 2a, b) indicating the presence of either a preceding instar or an embryonic cuticle.

The chela is held in front of the mouth opening (Fig. 2d, f). Both chela fingers are distinctly curved, and their pointed tips are crossed when closed (Fig. 2f). The immoveable finger is approximately as long as the chela palm from which it originates. The moveable finger (= ultimate cheliphore article) is more prominent than its immovable counterpart (Fig. 2f); it inserts laterally at the distal end of the palm, opening and closing along a medio-lateral plane. Near the base of each chela finger, the chela palm is equipped with a dorsal seta (Fig. 2f). Neither finger bears a (sub)terminal pore at its tip (Fig. 2g) and nuclear staining does not provide evidence for dense cell agglomerations in the chela. Both observations indicate a lack of chela glands.

The small palpal and ovigeral larval limbs are located lateral to the proboscis (Figs. 2c, d and 3a-c). Both limbs are composed of three podomeres, the distal-most being claw-shaped (Figs. 2d and 3b). However, owing to their delicate cuticle and the apparent lack of well-developed pivot joints, external delineation of these podomeres is difficult, especially in the case of the ovigeral larval limb. Nuclear staining shows labeling along the entire length of both limbs (Fig. 2c, e), confirming the presence of limb tissue underneath the cuticle (compare below). The palpal limb is slightly longer than the ovigeral limb and features a small posterior protrusion towards the distal end of its proximal podomere (Figs. 2d and 3b). A prominent seta on the proximal podomeres is absent (Fig. 2d).

Three walking leg anlagen are externally visible, exhibiting a pronounced antero-posterior (a-p) developmental gradient (Figs. 2c and 3a, b). In the most advanced walking leg 1, eight podomeres (coxae 1–3, femur, tibiae 1 and 2, tarsus-propodus precursor, main claw) can be externally delineated based on cuticular constrictions (Fig. 3c). No sign of a subdivision of the tarsus-propodus precursor into two separate podomeres is externally evident, and nuclear staining did not indicate an internal subdivision either (Fig. 2c). Coxa 3 and tibia 2 bear a small ventral seta-like outgrowth at their respective distal end (Fig. 3b, c). The tarsus-propodus precursor features either a single or two ventral spines along the future sole of the propodus (Figs. 2b and 3c). Additionally, three dorsal setae are consistently located distally (Fig. 3e, f). While the main claw of some specimens bears two seta-like outgrowths proximally (reminiscent of the prospective auxiliary claws) (Fig. 3e), only a single outgrowth in this position is found in other specimens (Fig. 3f).

Walking leg 2 is an elongate limb bud that curves medially towards the posterior body pole (Fig. 3a, b). Consistent external signs of articulation are still lacking, but nuclear staining reveals several (precursor) podomeres compressed within the cuticle (Figs. 2c and 3a). Owing to this compression, reliable identification of the podomeres is challenging and depends also on their developmental stage at the time of fixation (but see Fig. 2c). Beneath the distal cuticle tip, the developing main claw is always pressed into the tarsus-propodus precursor in accordion-like manner. Externally, the only reliable landmarks of the future podomere borders in this area are a ventral seta-like outgrowth at the future tibia 2-tarsus border, and a dorsal seta-like protrusion at the prospective border of propodus and main claw (Fig. 3d).

Walking leg 3 is a small external elevation flanking the posterior body pole with its ventrally protruding anal tubercle (Figs. 2c and 3b, d). Internally, the developing tissue of the leg is extremely compressed and folded, and identification of prospective podomeres or any kind of p-d regionalization is not yet possible (Figs. 2c and 3a).

The anlage of walking leg 4 is externally not recognizable. However, nuclear staining reveals its interior primordium in form of a denser accumulation of cells between the walking leg 3 elevation and the anal tubercle (Figs. 2c and 3a).

The anal tubercle lacks a functional anal opening (Fig. 3d) but the slit-shaped proctodeum is already discernible below the cuticle (Figs. 2c and 3a).

The dorsal side of the trunk lacks external segmentation lines (Fig. 2b, c). In contrast, the ventral side exhibits intersegmental folds that are especially pronounced between walking leg segments 1 to 3 (Fig. 3a, b). Nuclear staining reveals a compact brain anterior to the proboscis and the anlagen of all ventral segmental ganglia with a distinct developmental gradient along the a-p axis (Figs. 2c and 3a). The ventral cuticle of the more advanced walking leg segments shows tiny, bilaterally paired pits (Fig. 3b) that are aligned with the ganglion anlagen (Figs. 2c and 3a). Their presence is very likely related to segmental invaginations (so-called “ventral organs”) in the underlying ventral neuroectoderm (see [36] for details). The instar’s body and especially the appendages are sparsely covered by the so-termed “Gabelborsten”, a mechanosensory seta type found in most pycnogonids. Bifurcating and trifurcating variants of this seta type are present on the same specimen, reaching 10–15 μm in length (Fig. 3g).

### *Pallenopsis hodgsoni* Gordon, 1938

**Fig. 4:**
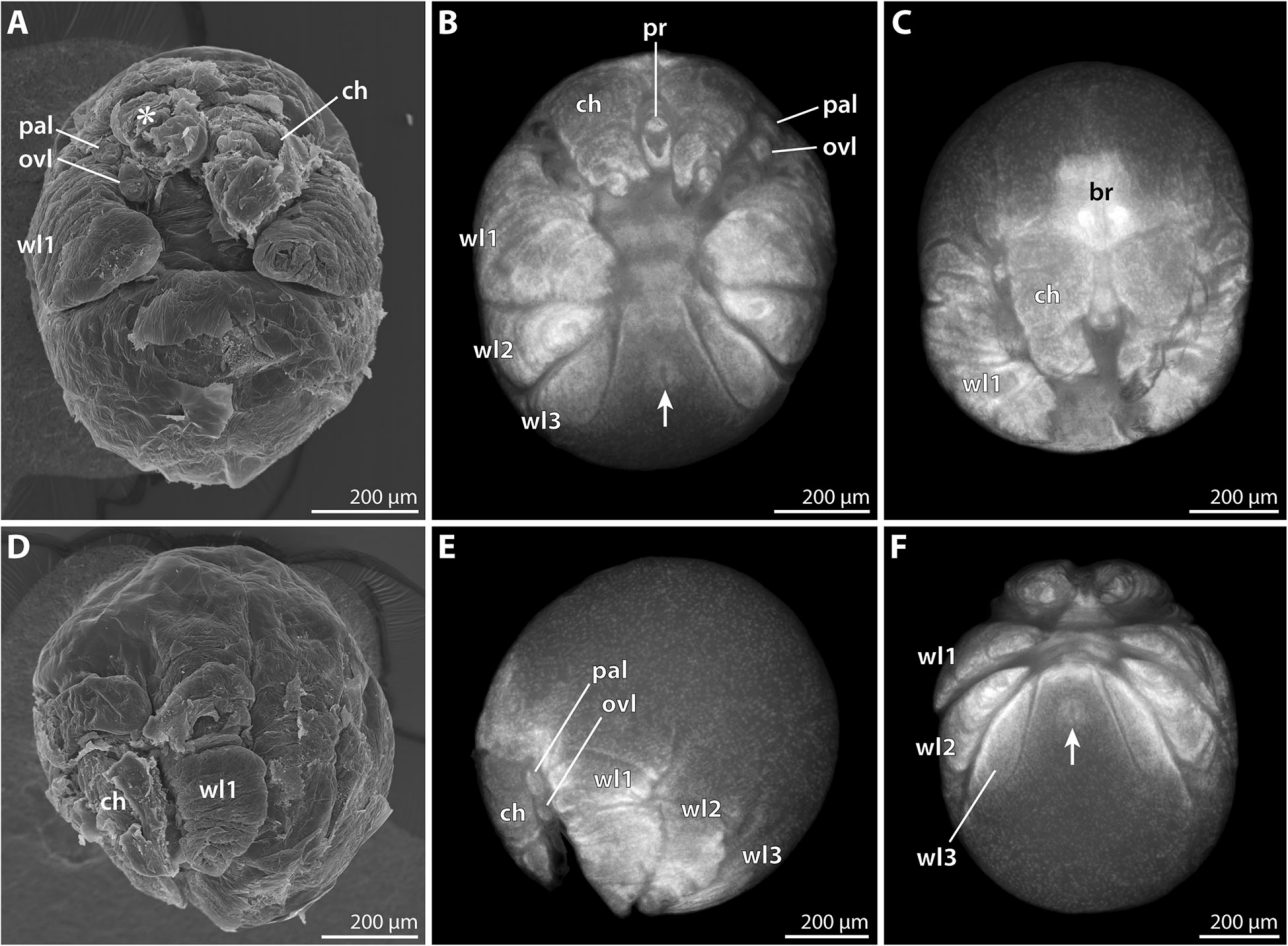
Prehatching embryo of *Pallenopsis hodgsoni*. SEM micrographs (**a**, **d**) and stereomicroscopic images of Sytox-stained specimens (**b**, **c**, **e**, **f**). The arrow points at the slit-shaped proctodeum anlage. **a** & **b** Ventral view. The asterisk in **a** marks the area in which the right cheliphore has been removed. Note the presence of walking leg pairs 2 and 3 underneath the embryonic cuticle. **c** Anterior view. **d** & **e** Lateral view. **f** Posterior view. Abbreviations: br – brain; ch – cheliphore; ovl – ovigeral larval limb; pal – palpal larval limb; pr – proboscis; wl – walking leg

**Fig. 5:**
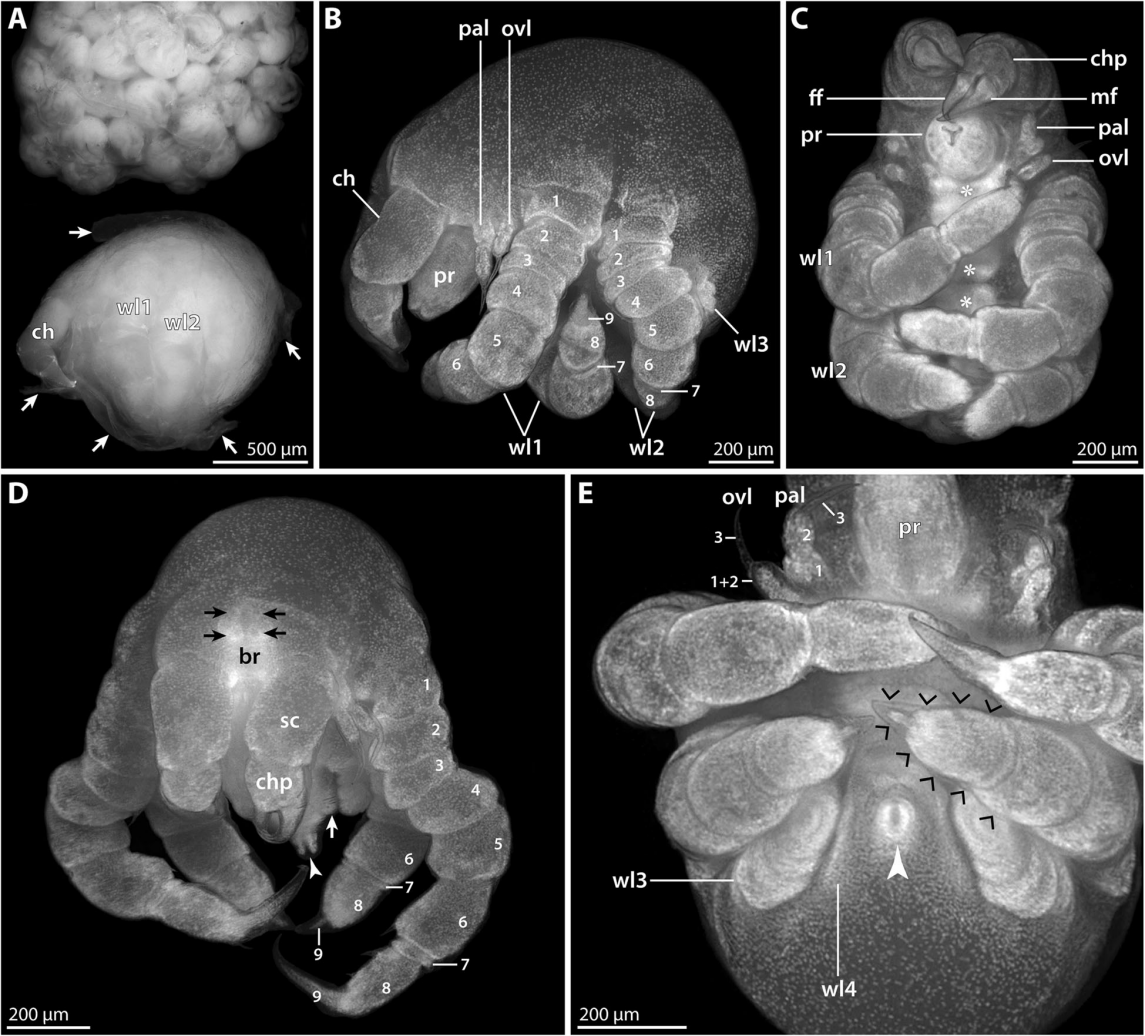
Postlarval instar 1 of *Pallenopsis hodgsoni*. Stereomicroscopic images of cuticular autofluorescence (**a**) and Sytox-staining (**b–e**). White arrowheads mark the slit-shaped proctodeum underneath the cuticle of the anal tubercle. **a** Upper half: overview of egg batch with still enclosed hatching instars. Lower half: hatching instar in lateral view. Arrows highlight the ruptured egg membrane. **b** Lateral view. Note the distinctly separated podomeres of walking legs 1 and 2. **c** Ventral view. Asterisks highlight the developing ventral ganglia. **d** Anterior view. Black arrows indicate the four eye anlagen. The white arrow points at the minute primordium of walking leg 4. The developing podomeres of tarsus (7) and propodus (8) are separated in walking leg 1 and 2. Note that the main claw (9) of walking leg 2 is still pressed into the propodus in accordion-like manner. **e** Detail in ventral view. The palpal larval limb features three distinct podomeres, whereas delimitation of the two proximal podomeres in the ovigeral larval limb is not straightforward. Small black arrowheads trace the unarticulated cuticle that covers the distal portion of walking leg 2. Abbreviations: br – brain; ch – cheliphore; chp – chela palm; ff – fixed chela finger; mf – moveable chela finger; ovl – ovigeral larval limb; pal – palpal larval limb; pr – proboscis; sc – scape; wl – walking leg

**Fig. 6:**
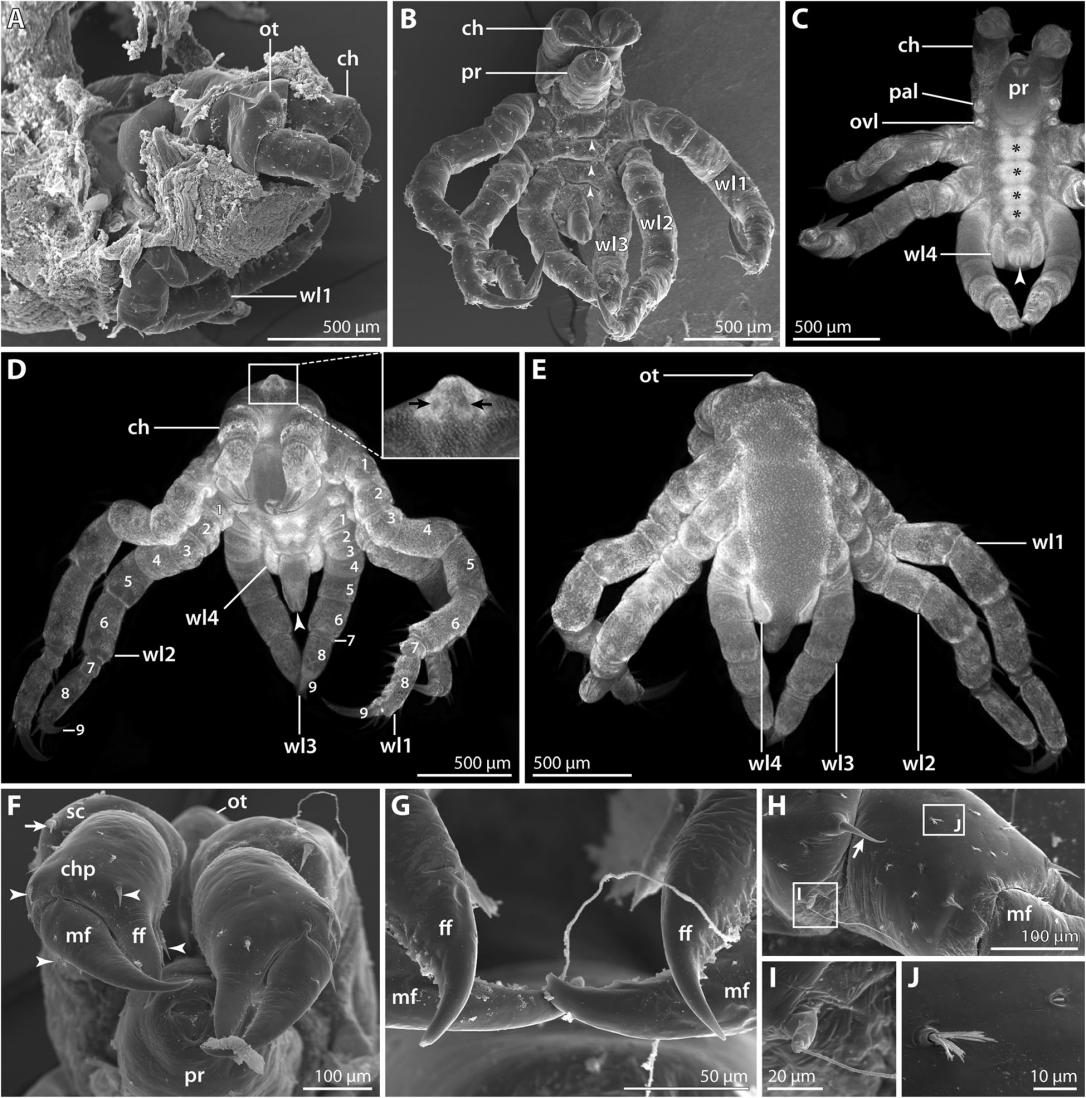
Postlarval instar 2 of *Pallenopsis hodgsoni*. SEM micrographs (**a**, **b**, **f–j**) and stereomicroscopic images of Sytox-stained specimens (**c–e**). **a** Specimen breaking through the egg matrix. Anterior to the right. **b** Ventral view. Arrowheads mark the ventral intersegmental folds between the walking leg segments. **c** Ventral view. Asterisks mark the developing ventral ganglia. The arrowhead points at the slit-shaped proctodeum. **d** Antero-ventral view. Walking legs 1 and 2 are fully articulated. Underneath the unarticulated cuticle of the elongate walking leg 3 limb bud, all nine podomeres are prefigured. The inset shows a magnification of the ocular tubercle with the anterior eye pair (arrows). The arrowhead points at the slit-shaped proctodeum. **e** Postero-dorsal view. Note the absence of any intersegmental folds. **f** Detail of the chelae. Arrowheads mark seta on the chela, the arrowhead points at the dorso-lateral seta at the distal end of the scape. **g** Detail of the chela finger tips which lack pores, indicating the absence of chela glands. **h** Detail of distal end of the scape and the chela palm. Lateral view. The arrowhead points at the dorso-lateral seta on the scape. Insets indicate the position of images shown in **i** and **j**. **i** Magnification of the single attachment gland process with emanating fibrous secretion. **j** Detail of slit organ (right) and a “Gabelborste” organized in two main bundles comprising numerous delicate setulae each. Abbreviations: ch – chela; chp – chela palm; ff – fixed chela finger; mf – moveable chela finger; ot – ocular tubercle; ovl – ovigeral larval limb; pal – palpal larval limb; pr – proboscis; sc – scape; wl – walking leg

**Fig. 7:**
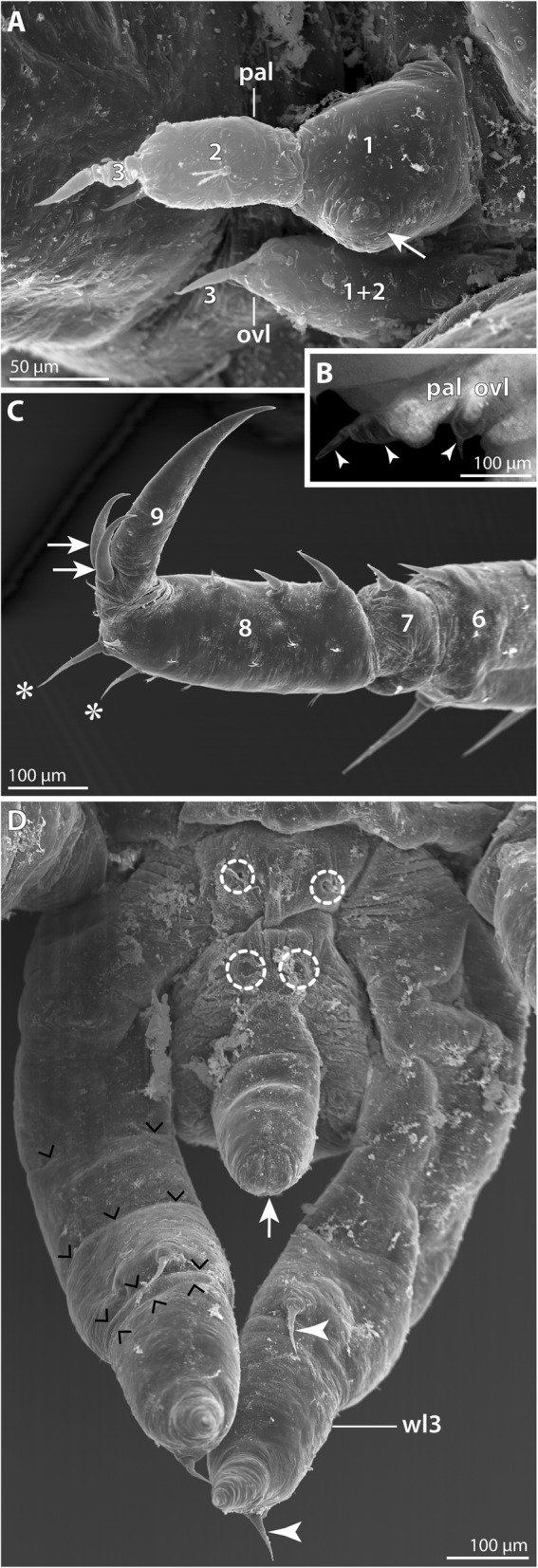
Postlarval instar 2 of *Pallenopsis hodgsoni* (continued). SEM micrographs (**a**, **c**, **d**) and stereomicroscopic image of Sytox-stained specimen (**b**). **a** Detail of palpal and ovigeral larval limbs. The arrow points at the posterior protrusion on the proximal article of the palpal larval limb. Note the externally undivided proximal article of the ovigeral larval limb (1 + 2). **b** Detail of palpal and ovigeral larval limbs. Arrowheads mark the outline of the external cuticle of both limbs. Note progressing atrophy of the limb tissues as indicated by the restriction of nuclear labeling to the proximal limb portions. **c** Detail of distal walking leg 2 podomeres. Asterisks mark two of the three stereotypic setae on the distal end of the propodus (8). Arrows point at the two auxiliary claws. **d** Detail of posterior body pole. Ventral view. The cuticle of the anal tubercle is distally closed (arrow). The stippled circles highlight the shallow cuticular pits of the ventral organs in walking leg segments 3 and 4. White arrowheads point at the characteristic ventro-distal seta of the future tibia 2 and the subterminal dorsal seta marking the distal end of the propodus. Black arrowheads highlight folds in the limb bud that correspond well with borders of internally developing podomeres. Abbreviations: ovl – ovigeral larval limb; pal – palpal larval limb; wl – walking leg

Two ovigerous males of *P. hodgsoni* were found in the CEAMARC 2007/2008 material (Table 1). One of them had both ovigers inserted into a single voluminous “egg” package containing predominantly the hatching postlarval instar 1 (Fig. 5a) but also few prehatching embryos (Fig. 4). This indicates a certain degree of developmental asynchrony within one package. The other male carried numerous specimens of postlarval instar 2 (Figs. 6 and 7).

#### Prehatching embryonic stage (SEM *n* = 1, nuclear stain *n* = 2; Fig. 4)

Only three specimens in this developmental stage were found embedded in the matrix of the “egg” package. For better documentation, the egg membrane surrounding the embryos was manually removed. At this stage, the embryos are slightly ovoid. Their elongate axis corresponds to the a-p axis and measures about 800 μm (Fig. 4a, b).

Nuclear staining reveals the majority of embryonic tissue to be located in one hemisphere (= ventral side), whereas the other hemisphere (= dorsal side) contains a copious amount of yolk that is covered by an unstructured thin layer of cells (Fig. 4b, e). The anlagen of proboscis, cheliphores, palpal and ovigeral limbs as well as walking leg pairs 1–3 are discernible, albeit considerably compressed. The walking legs show a distinct a-p developmental gradient (Fig. 4b, e, f), but due to their compression their exact structure remains unclear. Anteriorly, the brain anlage is found (Fig. 4c) and cell agglomerations of the developing ventral ganglia appear as more intensely labeled ventral areas between the walking legs (Fig. 4b). Further, the posteriorly forming proctodeum is visible between the anlagen of walking leg pair 3 (Fig. 4b, f). Primordia of walking leg pair 4 are not yet apparent.

In the single specimen studied with SEM (Fig. 4a, d), part of one cheliphore was removed to show the underlying structures. Yet, owing to the presence of at least one embryonic cuticle and hardened peri-embryonic liquid, the achieved resolution remained suboptimal. In spite of this, the SEM observation confirms that the palpal and ovigeral larval limbs lie squeezed between and partially covered by cheliphore and walking leg 1 (Fig. 4a-c, e). Further, the limb bud of walking leg 1 is ensheathed by its separate embryonic cuticle, setting it off from the more posterior body region with its undivided cuticle, which covers the anlagen of walking leg pairs 2 and 3 (Fig. 4a, d).

#### Postlarval instar 1 (nuclear stain *n* = 20); Fig. 5; Table 2)

This instar hatches from the egg membrane (Fig. 5a) and simultaneously sheds the embryonic cuticle. It remains embedded in the elastic matrix that has kept the eggs in a compact package (Fig. 5a).

The developmental stage of the hatching instar of this species corresponds in many aspects to the postlarval instar 1 described for *P. villosa* (Table 2). However, apart from these similarities, the present instar shows several deviating features: It is smaller, measuring only 1–1.2 mm along the a-p axis. The proboscis is bullet-shaped, i.e., it starts halfway along its length to taper gradually toward the distal mouth (Fig. 5b). The chela fingers are not evenly curved along their entire length, their proximal portions being instead rather straight (Fig. 5c, d). The size difference between the palpal and ovigeral limbs is more pronounced (Fig. 5b). While the three-articled structure of the longer palpal limb is readily discernible with nuclear staining, unambiguous delineation of the proximal two podomeres is in most cases not possible for the ovigeral limb (Fig. 5b, e). In walking leg 1, the adult number of nine leg podomeres is easily recognizable with nuclear staining, i.e., tarsus and propodus are at this stage well-defined, although both podomeres are externally still covered by an undivided cuticle (Fig. 5c-e). Also in the elongate limb bud of walking leg 2, the full number of podomeres can be recognized internally, being externally covered by an unarticulated cuticle (Fig. 5b-e). The main claw of walking leg 2 is still pressed into the propodus in accordion-like manner (Fig. 5e).

#### Postlarval instar 2 (SEM *n* = 8, nuclear stain *n* = 9; Figs. 6 and 7; Table 3)

This instar emerges from the egg matrix (Fig. 6a). It is still lecithotrophic and remains attached to the father’s oviger. Linear body length measurement resulted in 1.2–1.4 mm (Fig. 6b, c, e), which represents a slight underestimation when considering the body’s curvature along the a-p axis.

**Table 3:** Similarities and differences between postlarval instar 2 of the pallenopsids studied

Instar 2	Species		
	*P. villosa*	*P. hodgsoni*	*P. vanhoeffeni*
Nutrition	?	lecithotrophic	lecithotrophic
Body length (mm)	?	**1.2–1.4**	**≥1.5**
Ocular tubercle	?	**dome-shaped**	**cone-shaped w/ pointed tip**
Eyes/eye anlagen	?	present	present
Proboscis	?	**gradually tapering, increasing toward tip**	**+/− cylindrical w/ tapering only in distal portion**
Cheliphore	?	3-articled	3-articled
Chela fingers	?	proximal portion +/− straight, then curving toward tip	proximal portion +/− straight, then curving toward tip
Attachment gland process	?	single & short	single & short
Scape setae	?	**1 distal & dorso-lateral**	**3–4 distal & evenly spaced along the dorsal side**
Chela setae	?	**1 dorsal & ventral to mf base**; 1 dorsal & 1–2 ventral to ff base	**2–4 along mf base**; 1 dorsal & 1–2 ventral to ff base
Palpal larval limb	?	externally 3-articled; **> > ovigeral limb**; atrophy of limb tissue	externally 3-articled **=ovigeral limb**; atrophy of limb tissue
Ovigeral larval limb	?	**externally 2-articled**; atrophy of limb tissue	**externally 3-articled**; atrophy of limb tissue
Walking leg 1	?	9-articled	9-articled
Walking leg 2	?	9-articled	9-articled
Walking leg 3	?	elongate limb bud; internally 9 articles defined	elongate limb bud; at least 8 articles internally defined
Walking leg 4	?	tiny elevation w/ compressed internal tissue	tiny elevation w/ compressed internal tissue
Walking leg 1&2 setae	?	fe, tb1 & 2 w/ dorso-distal setae; **only tb2 w/ 1 dorsal seta** **at mid-length**	fe, tb1 & 2 w/dorso-distal setae; **tb1 & 2 w/ at least 3 dorsal setae at mid-length**
Anal tubercle	?	w/ slit-shaped proctodeum; anus not open yet	w/ slit-shaped proctodeum; anus in some specimens open
Ventral side of trunk	?	segmentation lines between wl segments	segmentation lines between wl segments
Dorsal side of trunk	?	no signs of segmentation	no signs of segmentation
Gabelborsten	?	**ca. 10 μm; multifurcating but arranged in two main bundles**	**20–30 μm;** **bi-, tri- or tetrafurcating**
Central nervous system	?	all ganglion anlagen well-defined	all ganglion anlagen well-defined

Diagnostic differences between species are highlighted in bold. In case of lack of or insufficient data (*P. villosa*), questions marks are shown

*Abbreviations*: *fe* femur, *ff* fixed chela finger, *mf* moveable chela finger, *tb* tibia; *wl* walking leg

At the anterior body pole, the dome-shaped elevation of the ocular tubercle has emerged, bearing two pairs of eyes, or their anlagen (Fig. 6d, e). Ethanol preservation precluded reliable assessment whether shading pigments are present at this stage.

The proboscis is directed antero-ventrally and tapers very gradually along its p-d axis. The tapering increases towards the proboscis tip with the Y-shaped mouth opening that is flanked by three cuticular lips (Fig. 6c, d, f). The lips are surrounded by an undivided cuticular ridge (Fig. 6f) which appears to be strongly sclerotized, as penetration of the nuclear counterstain into the underlying tissue was found to be less reliable (Fig. 6c, d).

The scape of the three-articled cheliphore inserts anterior to the proboscis (Fig. 6b, c, f). Distally, it bears one short attachment gland process from which a secretion fiber protruded in some, but not all specimens studied (Fig. 6h, i). Further, it features one prominent dorso-lateral seta (Fig. 6f, h).

The chela has started to assume a “pallenopsid-like” shape and orientation (Fig. 6d, f): the elongate palm is directed postero-ventrally, its distal portion bent outwards. The tips of the medially angled chela fingers are located in front of the mouth opening (Fig. 6f). Distally, the palm bears a dorsal and a ventral seta at the base of the moveable finger (Fig. 6f). A single dorsal seta is located at the immovable finger’s base, whereas one or two larger setae are found ventrally (Fig. 6f). The tips of both fingers cross when closed, but which finger comes to lie closer toward the mouth varies (compare Fig. 6f & g). Pores are lacking from the finger tips (Fig. 6g).

The size difference between palpal and ovigeral larval limbs has become more pronounced (Figs. 6b and 7a). The palpal limb has retained its external subdivision into three podomeres. However, nuclear staining shows that atrophy of the limb tissue has started, as the cuticles of the claw-like distal podomere and major parts of the second podomere are empty shells without tissue (Fig. 7b). The ovigeral limb is an unarticulated bud, bearing the small remainder of the distal claw (Fig. 7a). At the tip of this limb, tissue is also atrophied (Fig. 7b).

Walking legs 1 and 2 are functional, fully articulated and much longer than in the preceding instar. As in the adult, tibia 2 is the longest podomere. Femur and tibiae 1 and 2 bear several setae, the three most conspicuous ones being located distally. The longest of them is in dorsal position, the other two flanking it on both sides (Additional file 1: Figure S1A, B). In addition, tibia 2 features a prominent dorsal seta halfway along its length and another one ventro-distally close to the tarsus (Fig. 7c; Additional file 1: Figure S1A, B). The tarsus is well-defined with a single seta/spine on its ventral surface (Fig. 7c). The propodus is only weakly curved with five (walking leg 1) or three (walking leg 2) proximo-distally decreasing sole spines (e.g., Fig. 7c). Further, it bears distally three setae (Fig. 7c) in the same array as in *P. villosa* (Fig. 3e, f). The main claw is almost as long as the propodus and only slightly curved with two auxiliary claws on its dorsal base (Fig. 7c).

Walking leg 3 is not functional yet. It is covered by an unarticulated cuticle that can show signs of the podomeres developing underneath (Fig. 7d). Internally, its level of differentiation is comparable to walking leg 2 of the preceding instar: all nine podomeres are delineated but the three coxae are fairly compressed, and the main claw is still pressed into the propodus in accordion-like manner (Fig. 6d, e). As in *P. villosa*, the approximate tibia 2-tarsus border is externally demarcated by a ventral seta on the future tibia 2, while the propodus-main claw border is landmarked by a dorsal subterminal seta on the future propodus (Fig. 7d).

The anal tubercle is directed postero-ventrally. In the specimens studied, the slit-shaped proctodeum (Fig. 6c, d) still lacks an open anus (Fig. 7d). The anal tubercle is laterally flanked by a shallow elevation of the walking leg 4 limb bud (Fig. 7d). Beneath the bud’s cuticle, intense nuclear labeling indicates extreme compression of developing limb tissue (Fig. 6c-e).

The trunk shows distinct ventral intersegmental folds between the four walking leg segments (Fig. 6b), whereas no signs of segmentation are detectable dorsally (Fig. 6e; Additional file 1: Figure S1A). The well-developed segmental ganglia of the ventral nerve cord have retained only a slight size decrease along the a-p axis. “Gabelborsten” and slit-organs are found on the surface of the entire body (Figs. 6f, h, j and 7a, c) with higher densities on the appendages. In low magnification, the ca. 10 μm long “Gabelborsten” appear to be bifurcating. However, higher magnification reveals them to be of a multifurcating type, being composed of two main bundles comprised of multiple delicate setulae each (Fig. 6j).

### *Pallenopsis vanhoeffeni* Hodgson, 1915

A single male bearing postlarval instars on its ovigers was present in the CEAMARC 2007/2008 material (Table 1). Among the instars, very few hatching or molting specimens were encountered.

#### Postlarval instar 2 (SEM *n* = 5; nuclear stain *n* = 9; Figs. 8 and 9; Table 3)

**Fig. 8:**
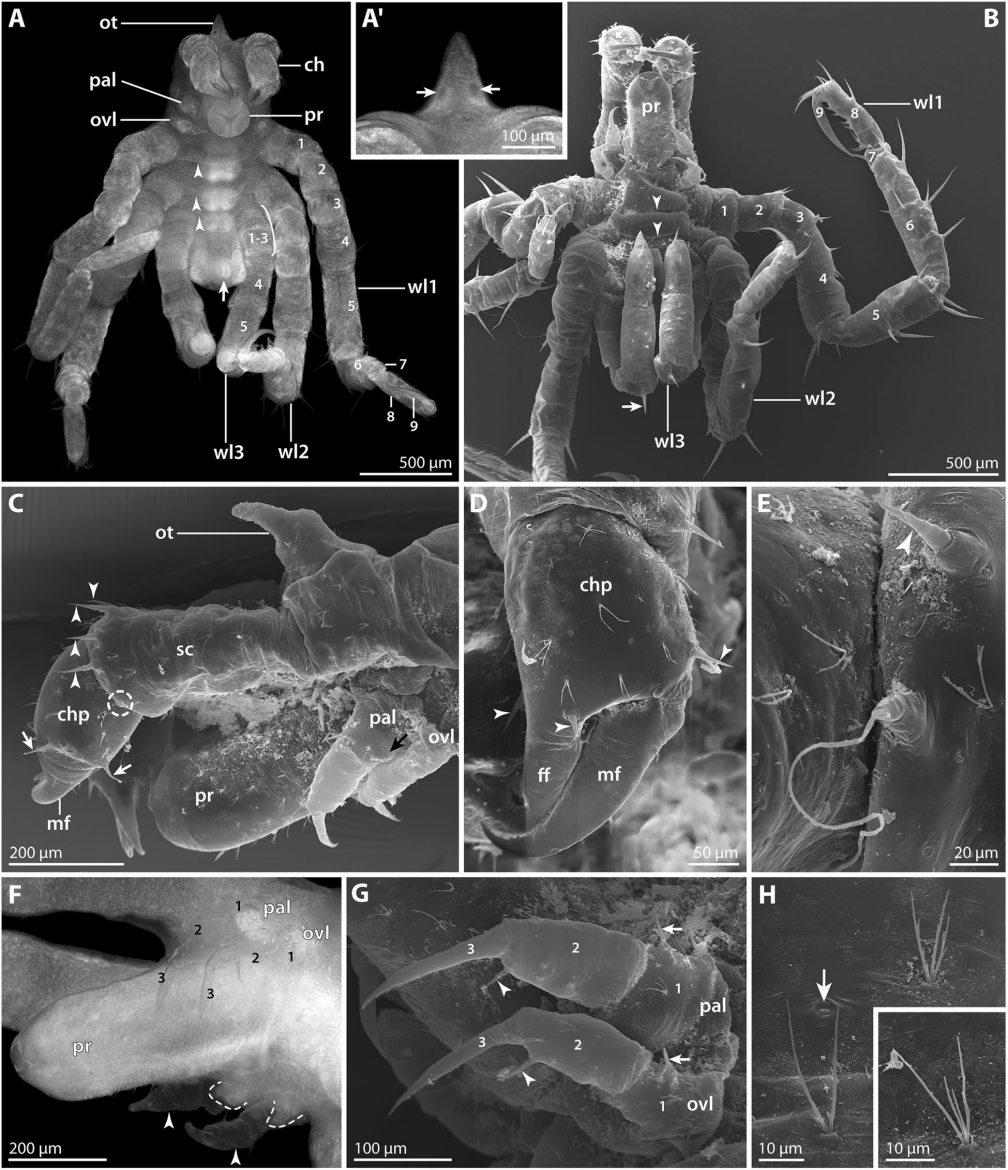
Postlarval instar 2 of *Pallenospis vanhoeffeni*. SEM micrographs (**B–E**, **G**, **H**) and stereomicroscopic images of Sytox-stained specimens (**A**, **A’**,**F**). **A** Ventral view. Arrowheads indicate ventral intersegmental folds. The arrow points at the slit-shaped proctodeum anlage. **A’** Detail of the pointed ocular tubercle. Arrows indicate the pair of anterior eyes. **B** Ventral view. Arrowheads indicate ventral intersegmental folds. Note the distinct dorso-distal seta on the future tibia 1 of walking leg 3 (arrow). **C** Anterior body pole. Lateral view. Arrowheads mark setae on the distal end of the scape. The stippled circle highlights the short attachment gland process. Arrows point at lateral setae on the chela palm. **D** Detail of the left chela. Arrowheads indicate characteristic setae at the base of the moveable and immovable chela fingers. **E** Detail of the attachment gland process. Note fibrous secretion emanating from the process of this specimen. **F** Detail of proboscis and larval limbs. Ventral view. Arrowheads point at the cuticular husks of the larval limbs, whereas the stippled lines indicate the extension of the remaining limb tissue. **G** Detail of the larval limbs. Note distinct external subdivision into three articles (1–3). Arrows point at the lateral seta of each proximal article. The arrowheads indicate the medial seta on each second article. **H** Details of bi-, tri- and tetrafurcating “Gabelborsten”. The arrow marks a slit-organ. Abbreviations: ch – cheliphore; chp – chela palm; ff – fixed chela finger; mf – moveable chela finger; ot – ocular tubercle; ovl – ovigeral larval limb; pal – palpal larval limb; pr – proboscis; sc – scape; wl – walking leg

The overall organization of this instar corresponds well to postlarval instar 2 of *P. hodgsoni* (Table 3). Despite the gross similarity, several characteristics allow ready distinction from the latter: The *P. vanhoeffeni* instar is larger, with a body length of 1.5 mm and longer walking legs. The more delicate structure of the walking legs and trunk resulted in fixation artifacts during ethanol preservation. This could be partially reversed in material rehydrated for nuclear staining, but not in SEM-processed specimens (e.g., ventrally collapsed walking leg pair 3 and anal tubercle in Fig. 8B).

**Fig. 9:**
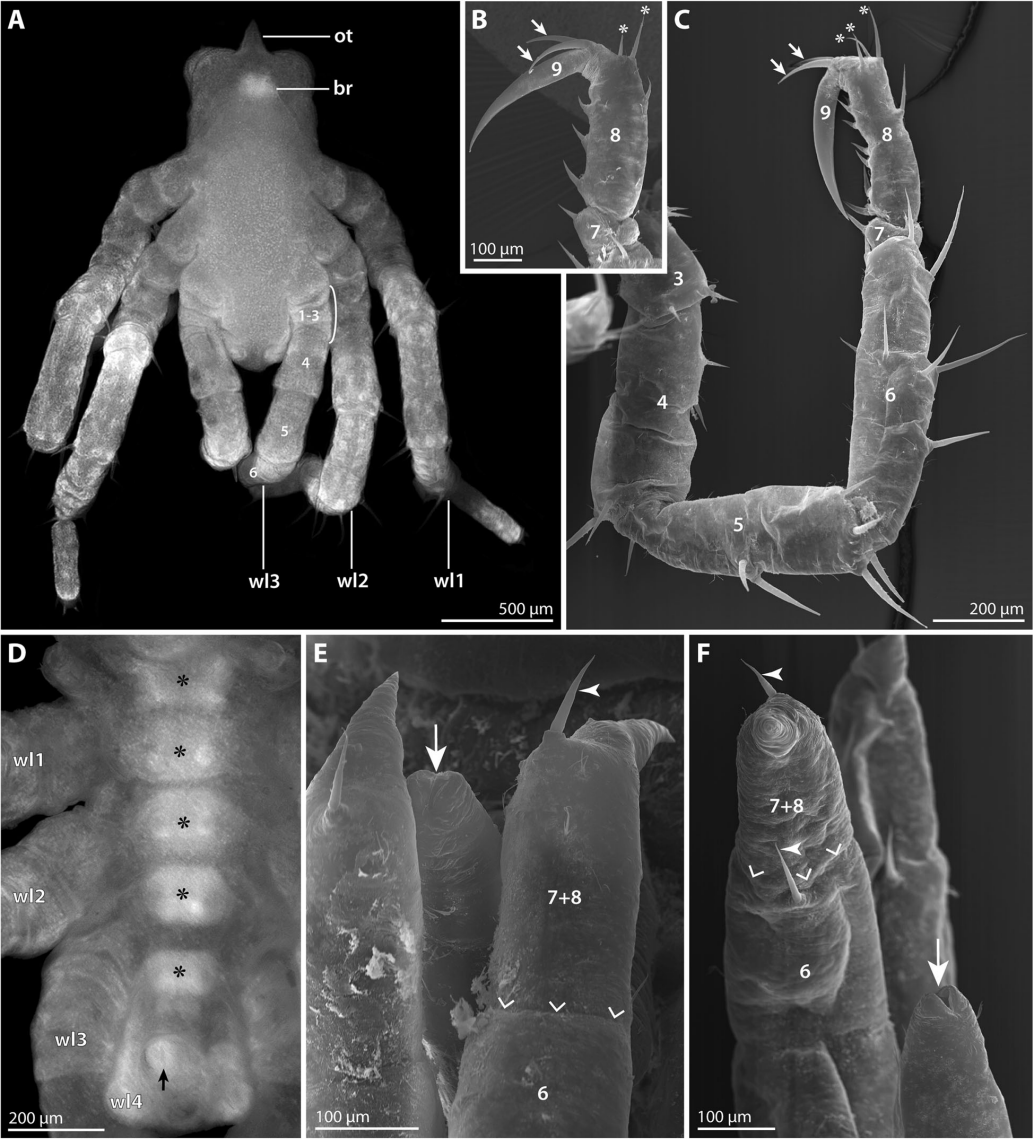
Postlarval instar 2 of *Pallenopsis vanhoeffeni* (continued). SEM micrographs (**b**, **c**, **e**, **f**) and stereomicroscopic images of Sytox-stained specimens (**a**, **d**). **a** Dorsal view. Note the absence of any intersegmental folds. **b** Detail of distal walking leg 2 podomeres. Asterisks mark two of the three stereotypic setae on the distal end of the propodus (8). Arrows point at the two auxiliary claws. **c** Walking leg 1. Note the long dorsal and dorso-lateral setae on the femur (4) and tibiae 1 and 2 (5 and 6, respectively). Asterisks mark the three stereotypic setae on the distal end of the propodus (8). Arrows point at the two auxiliary claws. **d** Detail of trunk. Ventral view. Asterisks mark the developing ventral ganglia. The arrow points at the ventrally directed anal tubercle. **e** & **f** Details of distal walking leg 3 limb bud and anal tubercle in two different specimens. The arrowhead points at the closed (**e**) and open (**f**) anus. The white arrowhead indicates the subterminal dorsal seta marking the distal end of the future propodus. In (**f**), another white arrowhead highlights the ventro-distal seta on the prospective tibia 2 (6). Small white arrowheads trace a fold that presumably corresponds to the future articulation line of tibia 2 and tarsus. Abbreviations: br – brain; ot – ocular tubercle; wl – walking leg

In comparison to *P. hodgsoni*, the longer and more numerous setae on cheliphore (Fig. 8C) and walking legs 1 and 2 (Figs. 8B and 9c) are striking. Further, the tall cone-shaped ocular tubercle is very conspicuous, tapering distally to a pointed tip (Fig. 8A, A’, C).

The proboscis is cylindrical along the major part of the p-d axis. Only its most distal portion tapers towards the mouth opening (Fig. 8B, C, F; Additional file 1: Figure S1C).

The dorsal side of the cheliphore’s scape is distally equipped with three to four long, evenly spaced setae (Fig. 8C). Ventral to the most lateral of them, a short attachment gland process is located. In some specimens, a secretion fiber protruded from this gland process (Fig. 8E, but Additional file 1: Figure S1D). As the instars were found to cling to the ovigers and egg matrix with their chelae and walking legs 1 and 2 (Additional file 1: Figure S1C, D), the gland’s secretion seems to serve a life line-like function rather than being the main form of attachment to the male. The chela palm bears an array of setae: two to four are found lateral to the base of the moveable finger, one is located close to the dorsal base of the immovable finger and one or two ventral to its base (Fig. 8C, D; Additional file 1: Figure S1C, D).

The palpal larval limb is slightly more robust but not significantly longer than the ovigeral one (Fig. 8C, G). Externally, both limbs are distinctly three-articled with a claw-like distal podomere. The proximal podomere bears a tiny lateral seta, whereas the second podomere is equipped with a medial one (Fig. 8G). Further, the proximal podomere of the palpal limb features a shallow posterior protrusion (Fig. 8C). Similar to *P. hodgsoni*, the limb tissues atrophy in this instar, resulting in the presence of almost completely empty cuticular husks in some of the specimens studied (Fig. 8F).

The nine-articled walking legs 1 and 2 bear prominent setae on all podomeres, out of which tibia 2 is the longest (Figs. 8B and 9c). Of the three coxae, only coxa 1 features a dorso-distal seta, while coxae 2 and 3 each bear three ventro-distal setae. Femur, tibia 1 and tibia 2 are equipped with several setae in ventral, lateral and dorsal positions, being located distally or halfway along the respective podomere (Fig. 9c). Without exception, the longest setae (up to 200 μm) are located dorsally. In some cases, their length even exceeds the diameter of the podomere from which they protrude (Fig. 9c). The short tarsus bears only a single ventral seta/spine, whereas the marginally curved propodus is armed with five to six (walking leg 1) or three (walking leg 2) spines along the ventral sole and a single subterminal dorsal seta plus three setae on its dorso-distal margin (Fig. 9b, c). The main claw is slender, only slightly curved and approximately as long as the propodus (Fig. 9b, c). At its dorsal base, a pair of auxiliary claws of about one third of its length are present.

Walking leg 3 is not functional yet. Along the p-d axis of the limb bud, indications of the interiorly differentiating podomeres can be recognized in some cases, but not consistently across all specimens. Nuclear staining shows that the proximally developing coxae 1–3 are still very compressed (Fig. 9a), whereas femur and tibiae 1 and 2 have already attained considerable extensions (Figs. 8A and 9a). A prominent dorsal seta marks the distal margin of future tibia 1 (Fig. 8B). Similar to *P. hodgsoni*, a ventral seta seems to demarcate the distal margin of tibia 2 (Fig. 9f), whereas a dorsal subterminal seta acts as a landmark for the distal margin of the future propodus (Fig. 9e). The anlage of the main claw is pressed into the latter in accordion-like manner.

In some specimens, the anus at the tip of the ventrally directed anal tubercle is open (Fig. 9f, but Fig. 9e), indicating that full functionality of the digestive system is reached during this instar and that the switch from lecithotrophic nutrition to active feeding will occur presumably after the next molt.

The entire surface of trunk and appendages is covered by slit organs and prominent “Gabelborsten” (e.g., Fig. 8C-E, H). The latter can be bi-, tri- or tetrafurcating, with lengths of 20–30 μm (Fig. 8H).

#### Hatching and molting stages (SEM *n* = 2; nuclear stain = 1)

Among the many postlarval instars attaching to the male’s ovigers, very few specimens were found to be in the process of hatching and/or molting.

**Fig. 10:**
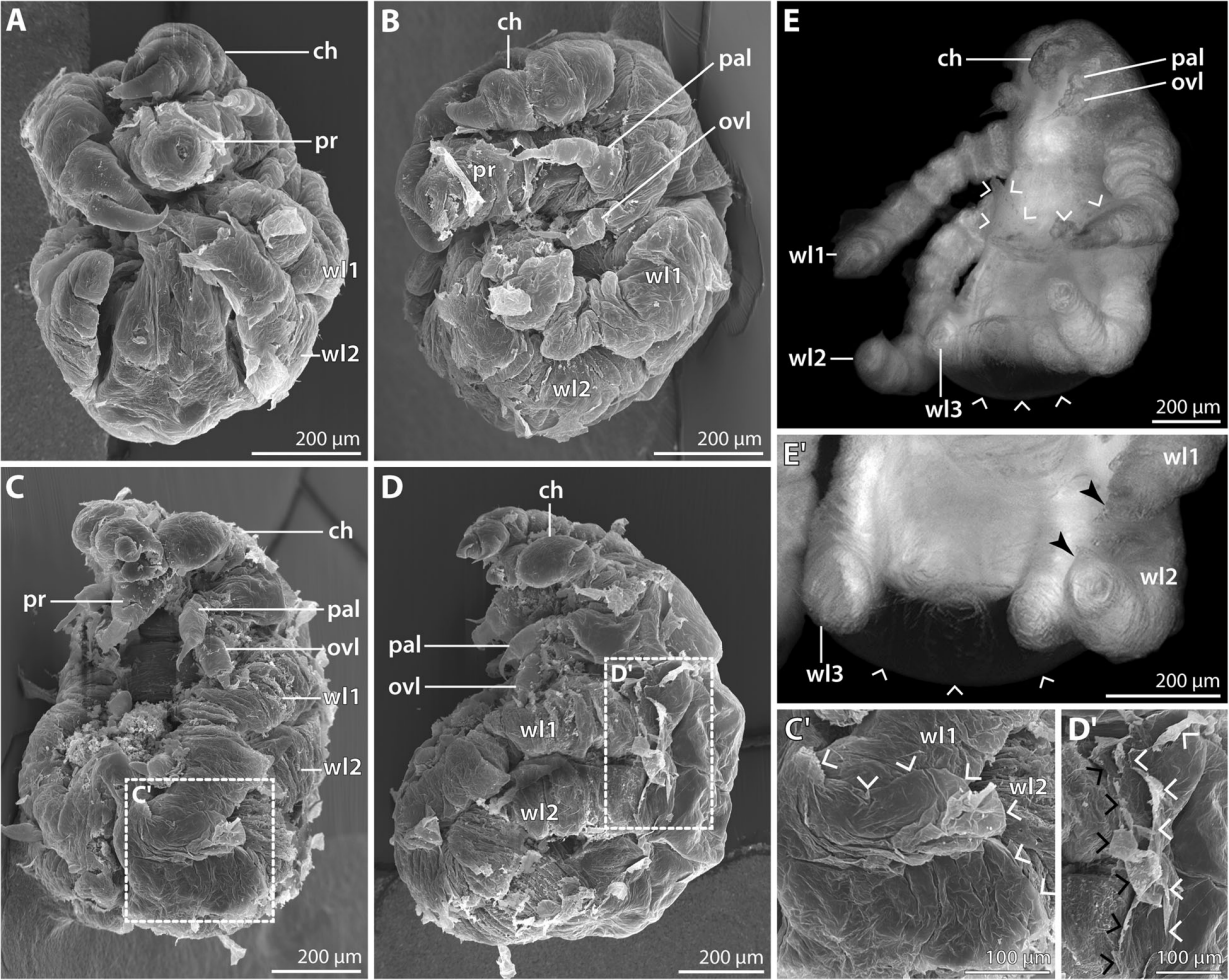
Prehatching embryos and presumptive postlarval instar 1 of *Pallenopsis vanhoeffeni*. SEM micrographs (**A–D’**) and stereomicroscopic images of Sytox-stained specimen (**E**, **E’**). **A** & **B** Prehatching specimen. Ventral (**A**) and lateral (**B**) views. Note pieces of at least one embryonic cuticle covering the proboscis tip and walking leg anlagen. **C** & **D** Hatching specimen. Ventral (**C**) and lateral (**D**) views. Note one or two additional cuticular layers that are still attached/being molted in some areas of the specimen (white and black small arrows in details **C′** and **D’**). **E** & **E’** Molting postlarval instar 1. **E** Ventro-lateral view. White arrowheads trace the cuticle that is being molted in the posterior body region. **E’** Detail of posterior body pole. Black arrowheads point at the tips of walking legs 1 and 2, their respective main claws remain pressed into the propodus. White arrowheads trace the cuticle that is being molted. Abbreviations: ch – cheliphore; ovl – ovigeral larval limb; pal – palpal larval limb; pr – proboscis; wl – walking leg

The earliest stage was manually peeled out of an almost complete egg membrane. It measures about 700 μm along the a-p axis (Fig. 10A, B). During membrane removal also pieces of at least one embryonic cuticle were unintentionally removed. In spite of the strong compression of most structures, the well-developed proboscis, cheliphores, palpal and ovigeral larval limbs, as well as the folded elongate anlagen of walking leg pairs 1 and 2 are discernible (Fig. 10A, B).

A second specimen is in a slightly more advanced stage of the hatching process (Fig. 10C, D). Here, the anlagen of walking leg pairs 1 and 2 have expanded and been pushed out ventrally, tearing two embryonic cuticles laterally along the body and close to the bases of the walking legs (Fig. 10C’, D’). The resulting presence of various fragments of different cuticular layers renders reliable assessment of further structural details by means of SEM challenging, calling for future histological and ultrastructural studies.

A last specimen appears to represent a hatched postlarval instar 1, which is already in the process of molting (Fig. 10E, E’; Table 2). The anterior appendages (proboscis, cheliphores, larval limbs) are unfortunately fairly collapsed (Fig. 10E), which is either an ethanol fixation artifact or indicative of an unsuccessful molt. In contrast, the posterior body features properly unfolded long walking leg pairs 1 and 2 and compressed limb buds of walking leg pair 3 (Fig. 10E). Judging by the cuticle that is being shed, neither walking leg 1 nor walking leg 2 have been fully functional prior to the molt, as a bona fide external articulation is missing. The main claws of both legs are still pressed into the propodus-tarsus (Fig. 10E’).

## Discussion

### Epimorphic tendencies in the development of Antarctic Pallenopsidae and other pycnogonid taxa

Very few previous studies mention details on the development of Pallenopsidae. To our knowledge, Carpenter [29] was the first to depict the hatching protonymphon larva of *Pallenopsis spinipes*, and it took more than a century until a second description of two *Pallenopsis* protonymphon larvae was published (*P. patagonica* and *P. yepayekae* in [30]). Although data on more advanced postembryonic instars of these three species are currently lacking, several features of the hatching larvae indicate that they follow the most common developmental pathway in extant pycnogonids (type 1 sensu [20]). Characteristic for type 1 development, the small protonymphon larva bears only three limb pairs (cheliphores + palpal and ovigeral larval limbs), leaves the paternal oviger, attaches to a host and undergoes anamorphic development with sequential differentiation of body segments.

Deviating from this pattern, the three Antarctic pallenopsid species studied by us exhibit lecithotrophic nutrition into far advanced instars in conjunction with extended attachment to the father’s oviger. Judging from the yolk amount that is still present in the midgut of the postlarval instars 2 of *P. hodgsoni* and *P. vanhoeffeni*, their attachment to the oviger will last at least up to the next molt, at which point they are going to be equipped with three functional walking leg pairs. Moreover, our data on these two species illustrate a pronounced embryonization of development, as the first three walking leg segments are fairly differentiated prior to hatching. Thus, regardless of gaps in the available series of developmental stages, these findings prove the presence of a novel developmental pathway for Pallenopsidae, which closely represents developmental type 5 sensu Brenneis and colleagues [20].

Outside of Pallenopsidae, extended embryonic development followed by lecithotrophic nutrition and prolonged attachment of postlarval instars (type 5 in [20]) is so far only known for Callipallenidae [25–28, 37] and some Nymphonidae [38]. Some authors have proposed to include Pallenopsidae in Callipallenidae (e.g., [32, 39, 40]); however, recent phylogenetic analyses do not support close affinities of the two groups (e.g., [41–43]). In light of this and when considering the small type 1 protonymphon larvae in other pallenopsid species, the here reported epimorphic tendencies and extended brood care are therefore most plausibly interpreted as an independently evolved, apomorphic trait within Pallenopsidae, potentially as an adaptation to cold water habitats (e.g., [44, 45]). A similar phenomenon is found in the ammotheid genus *Ammothea*, in which large lecithotrophic instead of free-living parasitic protonymphon larvae occur in several Antarctic representatives [14, 16, 18].

One interesting difference between Pallenopsidae and Callipallenidae is the differentiation of the palpal and ovigeral larval limbs during development. During the embryonic development of all callipallenids studied, palpal and ovigeral larval limbs are never fully differentiated and are often completely missing in the hatching instars [25–28, 37]. In the Antarctic pallenopsids, the three-articled larval limb pairs are first formed and then gradually atrophy during the postembryonic phase, as is the case for species with type 1 protonymphon larvae and free-living parasitic instars. However, the considerable body size and mass of the lecithotrophic pallenopsid instars renders their tiny larval limbs useless for grasping and attachment to any substrate. Accordingly, the persisting formation of such functionally inept limbs in the Antarctic pallenopsids may be indicative of a relatively recent shift of parts of an anamorphic postembryonic development into the embryonic phase.

In similar fashion, the presence of at least two embryonic cuticles as observed in *P. vanhoeffeni* may point to a relatively recent embryonization event among Antarctic pallenopsids. In contrast, investigated members of the exclusively direct developing Callipallenidae form only one embryonic cuticle that is shed during the hatching process (e.g., [25, 27]). Interestingly, Meinert [46] mentions for different Nymphonidae with large lecithotrophic instars that they are still wrapped in several cuticles after hatching. The co-occurrence of small parasitic, anamorphic developers vs. large lecithotrophic developers with epimorphic tendencies in Nymphonidae could indicate a relatively recent onset of embryonization in this group, comparable to that in Pallenopsidae. Yet, the close but unsatisfactorily resolved phylogenetic relationships of Nymphonidae with Callipallenidae [41–43] currently hamper reliable character polarization in this assemblage.

### Can details on pycnogonid life history add further insights for biogeographical studies?

Over the last decade, an increasing number of phylogeographical projects have started to scrutinize pycnogonid species/species complexes with wide distribution ranges in Antarctic and Subantarctic waters, often via a combination of morphological and molecular approaches. So far, available studies include representatives of Nymphonidae [3, 11, 47, 48], Colossendeidae [6, 7, 9, 12], Callipallenidae [13] and also Pallenopsidae [8, 10, 49]. This has not only led to a better understanding of the genetic diversity and substructure of different populations in the SO, but also resulted in the delimitation of previously unrecognized species. However, developmental characteristics of the pycnogonid species/species complexes have been given almost no attention. For some taxa, this is due to the fact that there are no data on their early development (e.g., Colossendeidae). But also aside from these cases, pycnogonids are very superficially subsumed as “brooders” (= males carry egg packages until hatching) and “crawlers” (= neither hatching stage, nor subsequent instars and adults are pelagic) with limited dispersal potential [1, 3, 13]. Yet, this generalization neglects that the hatching stages and their subsequent postembryonic development vary between different species.

The Antarctic pallenopsids studied herein possess some of the largest—if not the largest ever—reported eggs and hatching stages of pycnogonids followed by a lecithotrophic phase of attaching postlarval instars. With this type of development, only far advanced ontogenetic stages with presumably at least three functional walking leg pairs leave the father. Accordingly, these species qualify as some of the most extreme examples of “brooders” and “crawlers” among pycnogonids. On the other hand, the small type 1 protonymphon larva of other pallenopsids [29, 30] implies its early abandonment of the father followed by anamorphic development on/in an invertebrate host. While most type 1 protonymphon larvae do not show specific structural adaptations that may facilitate floating and dispersal in open water, exceptions are known in the form of flagelliform distal limb extensions or lamellae/dense setae fields on larval limb articles [45, 50–52]. Even without specialized structures, larval dispersal via open water has been suggested based on the observation that hosts infested with larvae of a species were frequently devoid of the respective adults (e.g., [53] for *Endeis spinosa*). This notion of some extent of larval open water dispersal becomes even more compelling for species with a life cycle in which adults and larvae do not even share the same sessile prey/host (e.g., *Pycnogonum litorale*).

In light of this, it is noteworthy that the morphologically variable *Pallenopsis patagonica* complex (and the now separated similar species *P. yepayekae*), with a distribution range from the Antarctic into lower latitudes along the South American coast (e.g., [10]), has been recently shown to feature a small type 1 protonymphon larva [30]. In contrast, the lecithotrophic and postembryonically attaching pallenopsids studied herein seem to show a more restricted distribution range in the SO [2]. As of now, this observation is only preliminary, and further studies are necessary to expand our incomplete understanding of the adult morphological and molecular diversity of Southern Hemisphere pallenopsids (e.g., [10, 49]). Nonetheless, potential correlations between distribution range and developmental type of species may help illuminating the dispersal potential of pycnogonid “brooders” and “crawlers” – which is key for a sound understanding of pycnogonid distribution and diversification patterns.

### Pallenopsid cheliphore development: Insights into the evolution of the first limb pair of Pycnogonida and Euchelicerata

In three of the four pycnogonid major taxa with fully developed adult cheliphores (Nymphonidae, Callipallenidae, Phoxichilidiidae), this first limb pair consists of three articles: (1) the proximal scape, (2) the palm with the protruding immovable finger and (3) the movable finger. Also in several pycnogonid groups that have small, non-chelate cheliphores with fewer articles or that even lack them in adults, atrophy of a three-articled chelate larval limb can be readily observed in late stages of postembryonic development (e.g., [51, 53–55]). Hence, the textbook sea spider is often depicted with a three-articled cheliphore as the “representative” state of Pycnogonida.

**Fig. 11:**
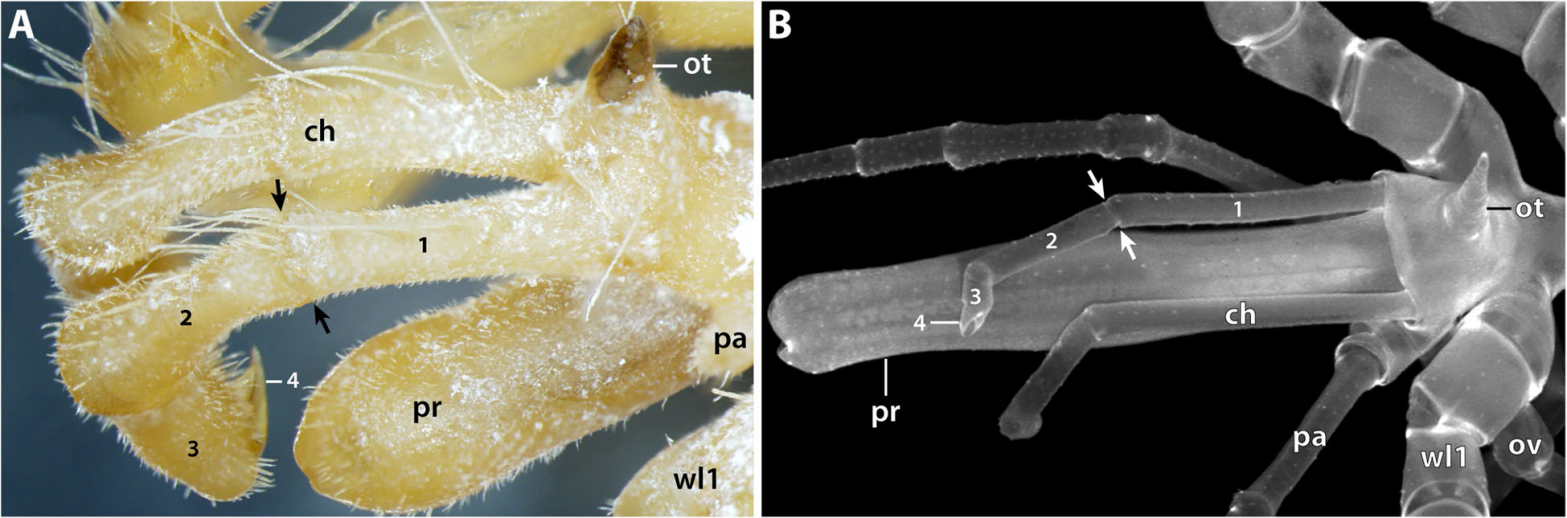
Four-articled cheliphores in Pycnogonida. Arrows mark the articulation line between the two scape articles. **a** Adult male of *Pallenopsis vanhoeffeni.* Dorso-lateral view of anterior body region. **b** Sub-adult specimen of *Colossendeis angusta*. Dorso-lateral view of anterior body region. Abbreviations: ch – cheliphore; ot – ocular tubercle; ov – oviger; pa – palp; pr – proboscis; wl – walking leg

However, several extant pycnogonids possess four-articled adult cheliphores with a two-articled scape. Examples are found in genera of Ascorhynchidae, Ammotheidae and Colossendeidae but also in Pallenopsidae (e.g., [41, 56, 57]). In fact, many species of the two pallenopsid genera *Pallenopsis* and *Bathypallenopsis* feature a two-articled adult scape, including the species studied here (Fig. 11a) [32, 38, 46, 58–60]. This is intriguing in light of fossil evidence, which proves four- and even five-articled cheliphores in the pycnogonid lineage [61–63].

Our data and the previous reports of protonymphon larvae in other pallenopsid species [29, 30] add new insights into the ontogenetic sequence leading to a four-articled adult cheliphore in extant Pycnogonida: pallenopsid development unequivocally demonstrates that the four-articled adult structure is preceded by a fully functional three-articled cheliphore in larval and/or postlarval instars. Hence, the four-articled adult limb has to arise via a subdivision of the proximal scape in more advanced (presumably juvenile) instars, similar to the subdivision processes of precursor podomeres in the differentiating walking legs (e.g., [15, 28]). Extrapolating this pallenopsid pattern to the pycnogonid crown group, the following scenario emerges: irrespective of a taxon’s adult condition, embryonic development of sea spiders leads to a functional three-articled cheliphore in the hatching stage (no exception known from any extant pycnogonid group). Notably, this developmental feature might even date far back into the pycnogonid stem lineage, as the cheliphore homolog of *Cambropycnogon klausmuelleri* – an early postlarval instar of a putative stem group representative from the Upper Cambrian – is short and potentially three-articled [64]. Any additional scape articles are then formed in more advanced instars during the anamorphic development that is widely assumed as being plesiomorphic for the pycnogonid lineage (e.g., [20]). Further, if using the Devonian *Palaeoisopus problematicus* with its three-articled scape (= five-articled cheliphore) as the hitherto most convincing adult stem lineage representative for character polarization [61, 62], such postembryonic scape subdivisions are resolved as plausible plesiomorphic feature of crown group development. Accordingly, the four-articled cheliphores in fossil [62, 63] and extant crown group members would be plesiomorphic, whereas three-articled adult cheliphores would result from an apomorphic truncation of the ontogenetic cheliphore segmentation program.

Importantly, however, phylogenetic studies on Pycnogonida show a disjunct distribution of crown group taxa with four-articled cheliphores [41–43, 65, 66]. This indicates multiple independent truncation events, if not even transitions in both directions. In light of the latter possibility, conclusive elucidation of the presence/absence of scape subdivisions in the crown group ancestor will have to rely not only on further fossil evidence but – somewhat paradoxically – also on developmental studies on extant groups lacking the structure of interest as adults: the taxa assumed to diverge near the base of the pycnogonid tree are the exclusively cheliphore-less Austrodecidae and Pycnogonidae and the predominantly cheliphore-lacking Colossendeidae [41–43]. While Pycnogonidae are a prime example of three-articled cheliphores prior to adult atrophy (e.g., [51, 52]), members of Colossendeidae still bear four-articled cheliphores as juveniles (Fig. 11b) and some genera even as adults (e.g., [41, 67]). Unfortunately, data on the embryology and early instars of Colossendeidae are missing, and the development of Austrodecidae, the putative sister group of all other extant pycnogonids [41, 42], remains completely unknown.

Beyond Pycnogonida, this scenario has also implications for the evolution of the chelate first limb pair in Chelicerata (Pycnogonida + Euchelicerata). The last common ancestor of crown group Chelicerata has been reconstructed with a three-articled first limb in adults [68, 69]. Yet, given the situation in the pycnogonid lineage, a reassessment of this view is justified, in particular when considering that some studies discuss a multi-articled raptorial first limb pair (the so-called “great appendage”) in the chelicerate stem lineage [70–72] (but see, e.g., [73, 74] for deviating views). Here, we propose such a multi-articled first limb (at least five articles) as plausible plesiomorphic adult state of the chelicerate crown group, which was then independently reduced in the pycnogonid lineage and the euchelicerate lineage by omission of segmentation processes during (postembryonic) scape/peduncle development. In spite of these considerations, pycnogonid development still supports the homology hypothesis of the three cheliphore articles formed during embryology (and retained in many pycnogonid adults) with the likewise embryonically formed three articles of the plesiomorphic adult chelicera in euchelicerates.

## Conclusions

Having been often directly preserved in high-percentage ethanol, developmental material of pycnogonids from remote oceanic regions is prone to show morphological fixation artifacts. In addition to the widely performed SEM study of external morphology (e.g., [16, 18, 19, 52]), fluorescent nuclear staining as used herein is an easy, inexpensive and time-effective way to (1) partially/completely reverse ethanol-induced collapse of external shape and (2) shed light on the differentiation of some internal structures (e.g., limb primordia, single leg podomeres and central nervous system). Hence, this approach, which is routinely applied to developmental stages of other chelicerate groups (e.g., [75]) has the advantage to provide more insights into developmental processes beneath the egg membrane and/or cuticle even in long-term stored pycnogonid material from museum collections.

Our study provides the first detailed description of pallenopsid development with epimorphic tendencies and extended attachment of advanced instars to the father. This novel type of pallenopsid development shows several correspondences to the more direct developmental pathways in some other pycnogonid taxa (Callipallenidae, Nymphonidae), which are most plausibly resolved as independently evolved traits in light of current phylogenetic hypotheses. In contrast to the rather undifferentiated categorization of pycnogonids as “brooders” and “crawlers” with very limited dispersal potential, we here suggest that different types of development may impact pycnogonid distribution ranges and encourage to include and test this notion in phylogeographical contexts. Further, pallenopsid cheliphore development provides interesting insights into the evolution of the raptorial first limb pair in Chelicerata. We propose a multi-articled, chela-bearing first limb pair as the adult condition of the last common ancestor of the chelicerate crown group, arising ontogenetically by postembryonic elaboration of a three-articled chelate limb that is formed during embryology.

## Additional file

Additional file 1: **Figure S1.** Stereotypic setae of the postlarval instar 2 of *Pallenopsis hodgsoni* (A, B) and *P. vanhoeffeni* (C, D). SEM micrographs. A: Walking legs 1 to 3. Dorsal view. Arrows exemplarily mark stereotypic setae on the distal ends of tibiae 1 and 2 (5 and 6, respectively). The white arrowhead points at the long dorsal seta halfway along tibia 2. B: Femur plus tibiae 1 and 2 of walking leg 1. Lateral view. Arrows indicate the characteristic dorso-distal seta and its flanking dorso-lateral seta on each podomere. The black arrowhead points at the dorsal seta halfway along tibia 2. The white arrowhead marks the ventro-distal seta of tibia 2. C: Anterior body region. Lateral view. Arrowheads mark two lateral setae on the chela palm. The arrow points at a long seta at the ventral base of the fixed chela finger. Note the section of egg matrix, which the chelae grab. D: Detail of chela. Lateral view. Arrowheads indicate three lateral setae on the chela palm (compare to C) and one seta at the dorsal base of the fixed chela finger. Arrows point at two setae at the ventral base of the fixed chela finger (compare to C). The short attachment gland process (stippled circle) lacks a fibrous secretion strand. Note that the chelae are used to grab pieces of egg matrix and strands of the secretion fibers for attachment. Abbreviations: chp – chela palm; ff – fixed chela finger; mf – moveable chela finger; ot – ocular tubercle; pr – proboscis; sc – scape; wl – walking leg. (TIF 4586 kb)

## Abbreviations

agp: attachment gland process
a-p: antero-posterior
br: brain
ch: cheliphore
chp: chela palm
ex: exuvia
fe: femur
ff: fixed chela finger
mf: moveable chela finger
ot: ocular tubercle
ov: oviger (segment)
ovl: ovigeral larval limb
pa: palp (segment)
pal: palpal larval limb
p-d: proximo-distal
pr: proboscis
sc: scape
SEM: Scanning electron microscopy
tb: tibia
wl: walking leg
