# Supplementary figures and images for: First description of epimorphic development in Antarctic Pallenopsidae (Arthropoda, Pycnogonida) with insights into the evolution of the four-articled sea spider cheliphore
Source: Zoological Lett. 2019 Jan 14;5:4. doi: 10.1186/s40851-018-0118-7 (PMC6330760; doi:10.1186/s40851-018-0118-7)

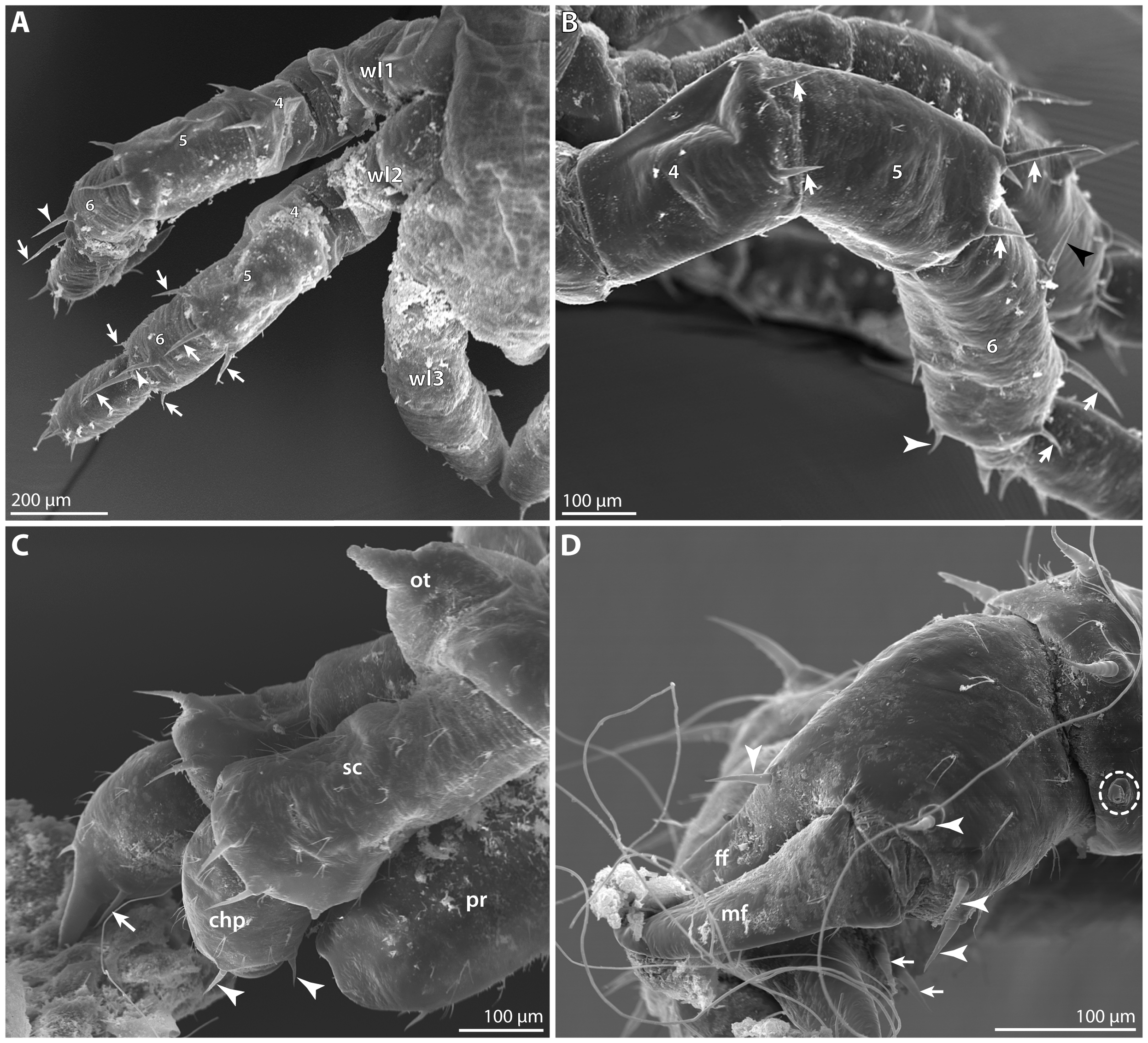

Supplement: Supplementary file 1 — Figure S1. Stereotypic setae of the postlarval instar 2 of Pallenopsis hodgsoni (A, B) and P. vanhoeffeni (C, D). SEM micrographs. A: Walking legs 1 to 3. Dorsal view. Arrows exemplarily mark stereotypic setae on the distal ends of tibiae 1 and 2 (5 and 6, respectively). The white arrowhead points at the long dorsal seta halfway along tibia 2. B: Femur plus tibiae 1 and 2 of walking leg 1. Lateral view. Arrows indicate the characteristic dorso-distal seta and its flanking dorso-lateral seta on each podomere. The black arrowhead points at the dorsal seta halfway along tibia 2. The white arrowhead marks the ventro-distal seta of tibia 2. C: Anterior body region. Lateral view. Arrowheads mark two lateral setae on the chela palm. The arrow points at a long seta at the ventral base of the fixed chela finger. Note the section of egg matrix, which the chelae grab. D: Detail of chela. Lateral view. Arrowheads indicate three lateral setae on the chela palm (compare to C) and one seta at the dorsal base of the fixed chela finger. Arrows point at two setae at the ventral base of the fixed chela finger (compare to C). The short attachment gland process (stippled circle) lacks a fibrous secretion strand. Note that the chelae are used to grab pieces of egg matrix and strands of the secretion fibers for attachment. Abbreviations: chp – chela palm; ff – fixed chela finger; mf – moveable chela finger; ot – ocular tubercle; pr – proboscis; sc – scape; wl – walking leg. (TIF 4586 kb) [file 40851_2018_118_MOESM1_ESM.tif]
